# Keep Calm and Do Not Carry-Forward: Toward Sensor-Data Driven AI Agent to Enhance Human Learning

**DOI:** 10.3389/frai.2021.713176

**Published:** 2022-01-12

**Authors:** Kshitij Sharma, Serena Lee-Cultura, Michail Giannakos

**Affiliations:** Department of Computer Science, Norwegian University of Science and Technology, Trondheim, Norway

**Keywords:** multi-modal data, sensors, educational technologies, learning analytics, motion-based games, AI agent

## Abstract

The integration of Multimodal Data (MMD) and embodied learning systems (such as Motion Based Educational Games, MBEG), can help learning researchers to better understand the synergy between students' interactions and their learning experiences. Unfolding the dynamics behind this important synergy can lead to the design of intelligent agents which leverage students' movements and support their learning. However, real-time use of student-generated MMD derived from their interactions with embodied learning systems (MBEG in our case) is challenging and remains under-explored due to its complexity (e.g., handle sensor-data and enable an AI agent to use them). To bridge this gap, we conducted an *in-situ* study where 40 children, aged 9–12, played MBEG on maths and language development. We automatically, unobtrusively, and continuously monitored students' experiences using eye-tracking glasses, physiological wristbands, and Kinect, during game-play. This allowed us to understand the different cognitive and physiological dimensions of students' progress (right/wrong responses) during the three different stages of the MBEG problem-solving processes, namely the “see-solve-move-respond” (S2MR) cycle. We introduce the novel Carry Forward Effect (CFE); a phenomenon occurring in such games, whereby students propagate, or “carry forward,” the cognitive and physiological effects derived from their MMD, to subsequent phases in the see-solve-move-respond cycle. By identifying moments when the Carry Forward Effect is congruent (or not) to students' learning performance, we uncover opportunities for feedback delivery to encourage or subdue the impact of the CFE. Our results demonstrate the importance of wristband and eye-tracking data as key indicators for prioritizing adaptive feedback to support students in MBEG and emphasize the significance of using MMD to support students' performance in real-time educational settings.

## 1. Introduction

Accurately assessing the cognitive and physiological processes underlying learning and play can enable researchers to understand the complex interactions occurring, system developers to design systems that account for those processes, and educators to scaffold the use of those learning systems (Clegg et al., 2017; Giannakos et al., 2020). Wearable and physiological sensors (e.g., eye-tracking glasses, smartwatches, wristbands, motion sensors) access data from students and enable us to capture their cognitive and physiological states (hereafter referred to as physio-cognitive states) in real-time. *Our goal is to extract the (near) real-time indicators (proxies) for physio-cognitive states and design a system to support and (potentially) enhance student's learning performance*^1^.

It is important to understand that the most salient physio-cognitive measures are responsible for human learning. Provided that such measurements can be computed and monitored in real-time, this advancement can help designers, developers, and educators proactively provide suitable feedback or scaffold students at appropriate times. Providing unsuitable feedback, or providing suitable feedback at inappropriate times, might have detrimental effects on students' performances (Schwartz and Bransford, 1998). Recent research has expressed much interest in the seamless integration of proactive and reactive support (e.g., presenting information to scaffold a student's problem-solving ability) to individual learning environments (Hattie and Timperley, 2007; Haapalainen et al., 2010; Wisniewski et al., 2020). The vision of this work is in alignment with Weiser's goals for the creation of environments saturated with sensing, computing, and wireless communication that gracefully support the needs of individuals and society (Weiser and Brown, 1997) (i.e., amplifying humans' learning capabilities, in our case).

Advances in mobile, wearable and sensing technologies, and the respective infrastructural developments, has enabled the automatic, unobtrusive, and continuous collection and synchronization of data from multiple sources (Sharma and Giannakos, 2020). Specifically, these sources empower us to collect attentional and cognitive (mobile eye-tracking glasses) motion (skeletal tracking) and physiological (electrodermal activity (EDA) and Heart Rate Variability (HRV) from wristbands) aspects of problem-solving. Moreover, analysis of such data provides a better understanding of the physiological (Di Lascio et al., 2018; Gashi et al., 2019; Mirjafari et al., 2019) and/or cognitive (Duchowski et al., 2018; Schaule et al., 2018; Gao et al., 2020) processes that underlie student performance, and also provide feedback to support their learning performance and interactions with technology (Liu et al., 2017; Sarsenbayeva et al., 2019). Our goal is to fuse physiological and cognitive information, extracted from wearable and ubiquitous sensing devices, for three distinct purposes: (1) to explain and predict learning performance; (2) to understand the physio-cognitive processes responsible for the different levels of learning performance; and (3) to design an intelligent agent that leverages sensing-based data to scaffold the learning processes.

To accomplish this, we collected and analyzed student's eye-tracking, motion and physiological (EDA, HRV) data, while they solved mathematical and English grammar problems [in the context of the motion based educational games (MBEG)]. We extracted multimodal measurements/features to explain and predict their learning performance and processes during various phases of problem-solving. The Multi-Modal Data (MMD) streams were analyzed to allow us to understand the key MMD-based indicators that are important to explain and differentiate between various levels of learning performance. We also present the design of an intelligent agent that leverages a combination of gaze, physiological-based measurements. The goal of the agent is to provide students with adaptive and seamless feedback, based on their physio-cognitive responses. To do so, we introduce two concepts that are central to the primary goal of the paper, the **See-Solve, Move, Respond (S2MR) phases** and the **Carry Forward Effect (CFE)**.

**See-Solve, Move, Respond phases** describe the three stages which occur during a student's interaction with a learning system. Problem-solving is initiated as the learning system prompts the student with a stimulus (e.g., question) to solve. The student *sees* and reads the question, mentally *solves* it, and visually identifies their desired response (See-Solve phase). Next, the student *moves* toward their computed answer and performs a physical action (i.e., gesture) to engage or select it (Move phase). Finally, the student *responds* to the question by providing their desired (right/wrong) answer to the agent as their response (Respond phase). Decomposition of this complete process constitutes one complete cycle of question presentation – solving – responding. This S2MR cycle re-starts each time a new question is delivered to the students and terminates once the students have provided their response. In this contribution, MMD was recorded during all three phases and was analyzed according to the S2MR phases in which it occurred, to explain/predict the learning performance levels and to select the most important MMD measurements from the aspect of performance prediction. The resulting selected measurements will be used to inform the design of the MMD-driven intelligent agent. One key advantage of the S2MR phases is that this division provides a generalizable sectionizing of a student's interactions with a learning system and can, therefore, be easily modified to fit any pragmatic definition of these phases in a particular learning system. Another advantage of defining the S2MR phases is the affordance of “early” predictions, which may provide the system with ample time to support struggling students or assist in preventing mistakes within a single problem solving cycle.

**Carry Forward Effect** describes the capacity of an MMD measurement/feature to explain/predict a student's learning performance across the different S2MR phases. If a measurement proves to be important (i.e., is highly associated with learning performance) across all three phases of the S2MR cycle, it is said to have a CFE. CFE acts as an early warning/predictor of students' performance. Early prediction of student behavior/performance has gained considerable traction in past research (Wolff et al., 2014; Hasan and Aly, 2019; Raga and Raga, 2019; Naseer et al., 2020). For example, Hasan and Aly (2019) used performance data from weekly quizzes and homework to predict students' final grades and researchers were able to identify students who were at risk of obtaining a low grade or course failure. Similarly, Raga and Raga (2019) used the click-stream data from an online course to predict student performance using a deep neural network architecture. Wolff et al. (2014) developed early prediction models to determine both dropout and failure probabilities in online courses. Recently, Naseer et al. (2020) also used click-stream data to predict collaborative performance using advanced machine learning algorithms in a software engineering course. Furthermore, several efforts in various domains have used machine learning methods for early prediction of student disengagement and dropout in an attempt to prevent the students from course withdrawal (Ruipérez-Valiente et al., 2017; Umer et al., 2017; Ortigosa et al., 2019; Cannistrà et al., 2020; Kemper et al., 2020). A common theme of the aforementioned early predictions research is to begin supporting students as early as possible during their interactions (or academic progression), to prevent adverse behavior which might impact, or correlate with, student's performance or engagement. For example, if a model is able to predict (with acceptable confidence) that a student will drop.out or perform poorly, then we can implement steps to prevent the occurrence of these events. These prediction efforts involve longitudinally collected data with similar long term dependent variables (e.g., dropout, at-risk students, low grades). To the best of our knowledge, there are limited short-term studies on early prediction (Lee-Cultura et al., 2020a), and other efforts are in a very specific context of intelligent tutoring system (Piech et al., 2015; Chen et al., 2018; Bhatt et al., 2020). Moreover, several studies employ methods that are based on complex algorithms which are difficult to describe (e.g., deep learning and hidden Markov models) and, thus, act as a “black-box” prediction of performance/engagement (aside a small number which use open learner models Badea and Popescu, 2020; Hooshyar et al., 2020; Somyürek et al., 2020). In turn, these studies do not directly relate to concrete design implications which can be leveraged for a scaffolding tool. With CFE, we aim for a systematic definition of measurements that can be easily monitored during short-term problem solving, while providing clear design guidelines to support struggling students. We provide both an inferential and predictive modeling approach to identify the measurements which might be detrimental to a student's learning performance, in a manner that is easy to understand for practitioners and designers alike.

The Carry Forward Effect is not an early detection of the relationship between a measurement and the wrong response. To detect whether a multimodal measurement displays CFE, all the phases are included from the presentation of the problem to receiving a response. First, the measurement has to be related to the correctness of the response in all the phases for it to be considered a CFE candidate. Second, the strength of the relation should decrease from the problem solving phase to the phases that are decreasingly less related to the problem solving. Once we establish that the measurement is related to the wrong response in all the phases and there is a slight decrease in the strength of this relation, we propose that remedial action is needed. It is not the case with all the measurements but the measurements that display CFE are the ones that should be considered important. Moreover, the behavior they (measurements that display CFE) serve as a proxy for should be scaffolded in a manner that the learning performance is improved. The core idea underlying this examination of measurements is that if there is a behavior that is so detrimental for learning performance that it has lasting trails into the non-problem-solving behavior, such behavior should be flagged and appropriate scaffolding should be provided to the students.

The main idea behind CFE is to provide prioritization for measurements to provide the feedback. We propose that the measurements that show CFE should be prioritized (to provide feedback accordingly) than those that do not show CFE. The main reason for this distinction is the fact that CFE extends to the non-problem-solving phases as well, showing the detrimental effect on learning (as our results suggest). Our proposal is that once the CFE-based measurements are taken care of by certain feedback mechanisms, only then the system should cater for other measurements.

In this paper, we show how CFE is determined using MMD collected from two games: suffizz and seaformuli. The main idea is that the students' interaction with the system is divided into phases: see-solve-move-respond. See and solve are the problem solving phases while move and respond are not related to the problem solving, *per se*. However, it is important to understand whether certain proxies for problem-solving behaviur (e.g., stress or cognitive load) are having their trails not only in the see-solve phases but also in the move and respond phases. In such situations, remedial actions corresponding to such behavior should obtain higher priorities than those who do not leave their trails in the non-problem-solving behavior.

With these two novel concepts (S2MR and CFE) in consideration, our work presents (1) empirical evidence that quantifies the relation between student's learner performance and MMD in real-time; (2) MMD measurements characterized by CFE; and finally, (3) implications for the design of an AI agent which leverages sensor data. Specifically, we investigate the following research questions (RQ):

1. How are a student's multi-modal measurements associated with their learning performance (correctness of their responses) during the different phases of the S2MR cycle?
2. How can multi-modal measurements inform the design of a physio-cognitive aware intelligent agent?

The contribution of this work is 3-fold:

- **Methodologically**, we use multiple data streams (eye-tracking, physiological, and kinematics data) to study the relationship between physio-cognitive behavior and performance in the context of MBEG.
- **Analytically**, we show the relation between a student's MMD measurements and their learning performance across the S2MR cycle (i.e., the interaction phases).
- **Conceptually**, we provide insights for the design of a physio-cognitive aware intelligent agent, derived from children wearing sensing technologies during MBEG play.

### 1.1. Theoretical Background: Scaffolding in Problem-Based and Game-Based Learning (GBL)

Scaffolding in problem based learning (PBL) plays an important role when the problems are structured (Reiser, 2004) or ill-structured (Hmelo-Silver, 2004). There can be a number of strategies to scaffold students during PBL. For example, enlisting interest (Belland et al., 2017), expert modeling, and question prompts (Van de Pol et al., 2010), and pointing toward important problem elements to consider (Wood et al., 1976). These scaffolds can affect the quality and correctness of the solution (Oliver and Hannafin, 2000). Janson et al. (2020) argue that scaffolding in PBL could be critical for the successful fostering of PBL, especially in technology-mediated environments. Taking the scaffolding a step further in technology-enhanced environments, Kim and Hannafin (2011) showed that the dynamic scaffolds provide a better interaction between the learners and the scaffolding source than the static scaffolds. Both procedural (e.g., step-by-step tutorials) and conceptual (e.g., providing hints or cues) scaffolds could help the learners in over-coming the learning challenges, provided they are supporting the learning and problem-solving processes, i.e., in a dynamic manner (Cagiltay, 2006; Way and Rowe, 2008). Sharma and Hannafin (2007) further argue for seamlessly integrating and balancing different scaffolds into the learning contexts. In line with Sharma and Hannafin (2007), Chen (2020) also suggests situated scaffolding to improve motivation and learning performance. Whereas, Haruehansawasin and Kiattikomol (2018) showed that such a scaffolding would be especially beneficial for the low-achieving learners. Janson et al. (2020) provide a systematic approach, adapted from Kim and Hannafin (2011), to scaffold learners in PBL by dividing the whole problem-solving process into five phases (i.e., engagement, exploration, explanation, justification, and reflection) and provide appropriate feedback in each of these phases. The positive effects of dynamic technology-mediated scaffolding are also highlighted in a meta-analysis by Kim and Hannafin (2011).

When it comes to GBL, scaffolding has been extended from a teacher (or more knowledgeable peer, Wood et al., 1976; Collins et al., 1991) to a software-based tool to support learners (Collins et al., 1991; Quintana et al., 2004). Similar to PBL scaffolding in GBL can both be procedural and conceptual. Recent results have emphasized the role of scaffolds in improving both the learning outcomes (Honey and Hilton, 2011; Garris et al., 2017) and learning experiences (Neulight et al., 2007; Broza and Barzilai, 2011) in GBL. In their review of scaffolding in digital GBL, Melero et al. (2011) found that such scaffolds promote positive attitudes along with positive effects on learning and highlighted the use of automatic tools (e.g., prompts, examples, hints, cognitive tools) in providing better scaffolds. The adaptive and fading nature of the scaffolds in such environments is highly important so that the learners can get support when they most require it (Ke, 2016). It was shown that the inherently dynamic interaction with the GBL environments might lead to trial-and-error behavior (Leemkuil and Jong, 2011). Therefore, it is important to design scaffolds that can support learners in a dynamic manner (Kao et al., 2017). However, the timing and type of scaffolds in such an environment should be carefully planned, as they can moderate the effectiveness of the support provided to the learners (Wouters and Van Oostendorp, 2013).

In this contribution, with CFE, we propose a MMD-based approach to combine and prioritize scaffolds while student are interacting with technology-enhanced problem-solving environments. We argue that it is important to provide scaffolding, especially in technology-enhanced learning environments, in a dynamic and stepwise manner (as shown by the recent work cited above). Previous theoretical and empirical contributions to both PBL and GBL have indicated toward a dynamic and stepwise scaffolding methods to be better than their static and overall counterparts. In our case, the “*See-Solve-Move-Respond” phases provide us with an opportunity to design dynamic scaffolds; whereas, the CFE provides an approach to combine and prioritize the type of feedback necessary at a given moment* in the problem-solving process. We use data from various sources not only to identify the different phases in the learners' interaction with the game but also to show which measurements during these interactions (e.g., cognitive load, stress, fatigue) are to be taken into consideration while providing support for the learners.

## 2. Related Work

In this section, we review contributions that assess performance using the individual gaze, physiological (EDA, HRV) and motion data streams used in our research. Additionally, we present the rationale behind the use of these streams by citing a collection of studies that have demonstrated great potential of MMD over individual constituent data sources, for measuring learning performance.

### 2.1. Gaze-Based Performance Assessment

Over the past few decades, gaze data has been used to assess performance in various scenarios. In the earlier years of eye-tracking technology, gaze data was primarily considered a research tool and used only in controlled lab studies. However, with the advent of mobile eye-tracking technologies, gaze data has established itself as an ecologically valid source. Concerning learning contexts, eye-tracking has been used in a number of educational domains and paradigms such as programming (Sharma et al., 2013), online and distance learning (Kizilcec et al., 2014), multimedia learning (Alemdag and Cagiltay, 2018), and GBL (Zain et al., 2011; Conati et al., 2013; Heinemann et al., 2020). For example, gaze-behavior was employed for evaluation purposes in GBL settings. Notably, adaptive hints during an educational game were shown to increase the students' performance, as well as the degree to which they paid attention to the hints (Conati et al., 2013). In a similar vein, eye-tracking data was also used to evaluate design decisions in serious games to augment student performance (Zain et al., 2011; Heinemann et al., 2020).

Additionally, eye-tracking data has been used to explain, understand, and monitor several learning processes, such as cognitive workload (Duchowski et al., 2018; Schaule et al., 2018), attention (Abdelrahman et al., 2019), mind-wandering (Hutt et al., 2019), information processing behavior (Sharma et al., 2020a), and fatigue (Rostaminia et al., 2017). Recently, off-the-shelf mobile eye-trackers have extended eye-tracking research beyond lab settings and into more ecologically valid educational settings. In this domain, mobile eye-tracking data has been used for skill-estimation of the students (Augereau et al., 2016) and to estimate the amount of attention students paid to their textbooks (Ishimaru et al., 2016). Mobile eye-trackers have also been used in informal learning settings, such as museums, to understand how students interact in exhibitions (Jung et al., 2018) and with their peers (Sharma et al., 2020b). Furthermore, mobile eye-tracking data has helped researchers understand students' collaborative behaviors in informal learning settings dependant on tangible user interfaces (Schneider et al., 2016).

Overall, gaze data has proven useful in explaining and predicting problem-solving performance and problem-solving behavioral patterns. Many of the findings conducted in stationary eye-tracking settings can be transferred to mobile and wearable contexts (i.e., using eye-tracking glasses). Therefore, in this contribution, we used mobile eye-tracking glasses to record students' gaze data. This data is used to model student's cognitive and attentional processes.

### 2.2. Physiological Data-Based Performance Assessment

There is a large body of research dedicated to performance assessment using physiological data (i.e., EDA and HR/HRV). These recent contributions utilize low-cost consumer-grade smartwatches (Goyal and Fussell, 2017; Schaule et al., 2018) and wristbands designed for research purposes (Gjoreski et al., 2018; Kosch et al., 2019) to explain or predict the performance of students (Rissler et al., 2018; Sharma et al., 2020c), drivers (Solovey et al., 2014), players (Tognetti et al., 2010; Huynh et al., 2018), and workers (Rissler et al., 2018; Kosch et al., 2019). For example, the direction of intensity change in phasic EDA was used to infer the performance of participants in a collaborative task (Goyal and Fussell, 2017). Features extracted from EDA and HRV have been used to monitor cognitive workload in conjunction with self-reports of NASA Task Load Index (NASA-TLX) (Gjoreski et al., 2018; Kosch et al., 2019). Moreover, significantly high correlations were found between workplace performance and heartbeat regularity (Mirjafari et al., 2019). By utilizing HRV features measured with Photoplethysmograph (PPG), Zhang et al. were able to classify cognitive workload with an accuracy of 97% and 87% during static and interaction testing, respectively (Zhang et al., 2018). In a recent contribution, features computed from EDA, HRV, Blood Volume Pulse (BVP), and skin temperature were used to predict the cognitive performance in various studies with a low error-rate (Sharma et al., 2020c).

Moreover, in educational contexts, there has been an increase in approaches that utilize physiological responses for gauging engagement, monitoring learning performance, and adapting learning difficulty. Di Lascio et al. (2018) used an Empatica E4 (Emp, 2021) physiological-monitoring wristband to assess students' engagement during lectures. In a follow-up work, “physiological synchcrony” was combined with EDA features to estimate the engagement between presenters and their audience (Gashi et al., 2019). Similarly, Radeta et al. (2017) used EDA measurements to compare two interactive learning experiences for children: a mobile game and animated storytelling. The authors were able to quantify and link learning for both experiences to EDA peaks. Furthermore, data-driven clusters, including EDA, were used to explain children's various construction activity strategies (Worsley and Blikstein, 2015). Lastly, EDA was used to monitor self-regulation strategies while students were answering a set of questions as part of their exams (Noroozi et al., 2019).

Overall, physiological data collected from wearable and sensing devices has proven to be transformative for tracking students' performance in different contexts. Our research investigates the extent to which physiological data can provide insights into students' learning in an accurate and timely manner. In doing so, we extend previous relevant studies and showcase the applicability of physiological data affordances in the context of intelligent learning systems.

### 2.3. Why MMD?

A holistic understanding of complex learning processes cannot be attained when only using individual data sources (Sharma et al., 2019a; Sharma and Giannakos, 2020). For example, eye-tracking and EEG do not provide students' affective information, while facial data, HRV, EDA, and similar physiological data sources lack cognitive and attentional aspects of these processes. The aforementioned data streams each provide knowledge regarding select aspects of students' learning processes and/or outcomes, but to gain a holistic understanding of the processes correlated to a student's learning performance (Giannakos et al., 2019; Sharma et al., 2019a), the fusion of information extracted from multiple data sources is necessary (i.e., MMD, Blikstein and Worsley, 2016). Research has shown that MMD provides better results, regarding students' performance prediction and behavior explanation. When combined, these data demonstrate synergistic relationships and provide researchers with a richness of information that is bigger than the sum of the components.

In numerous studies, predictive performance models containing fused data sources have outperformed predictive performance of the individual data sources (Cukurova et al., 2019; Giannakos et al., 2019; Liu et al., 2019; Sharma et al., 2019b, 2020c; Lee-Cultura et al., 2020a). For example, one study which used a modified-Pacman game, found that the fusion of EEG, eye-tracking and facial data streams, outperformed the individual data streams when predicting player performance (Giannakos et al., 2019). Similarly, in an adaptive self-assessment test, the combination of eye-tracking, facial features, EDA, and HRV data showed lower error rates than the individual components when predicting engagement and performance (Sharma et al., 2019b). In the same vein, combining features from eye-tracking, motion, EDA and HRV have resulted in better performance prediction during children's play with MBEG, than the individual data stream (Lee-Cultura et al., 2020a). Lastly, in a diverse set of studies (e.g., games and learning tasks), the combination of facial data, EDA, BVP, and HRV resulted in a lower error rate while predicting participant's cognitive performance when compared against the error rate achieved by the individual features (Sharma et al., 2020c).

The prevailing advantages of MMD over individual data streams demonstrated in empirical studies also extend to collaborative cases (Olsen et al., 2020; Vrzakova et al., 2020). This shows a synergistic fusion of information when individual data sources are combined, which results in higher predictive quality from MMD. However, despite the indicated advantages of using user-generated MMD to understand and predict students' learning experiences, as well as the affluence of using MBEG to amplify students' learning (Retalis et al., 2014; Tsai et al., 2015; Kourakli et al., 2017; Chang and Tsai, 2018; Lee-Cultura et al., 2020b), little research on wearable and physiological sensors has been conducted in the domain of maths and language based MBEG. For this reason, we combine data from mobile eye-tracking, motion capture, and wristbands to explain and predict performance in MBEG.

## 3. The Motion-Based Educational Games

In this section, we provide a detailed account of the two MBEG used in our study, Suffizz (which centers on English language competence) and Sea Formuli (which targets arithmetic competence). In both games, the student is presented with a series of 5 multiple choice style fill-in-the-blank problems, each with 3 potential answers. The games are Kinect-based (Zhang, 2012) and transform the student into a living cursor through the use of gesture (to select on-screen items) and full-body movement (to relocate selected items). Students select an item by performing a grabbing gesture and maintaining a closed fist on the item. They move the selected item by repositioning their body in physical space and dragging their fist through the air toward their desired on-screen destination. In this way, the games offer the same affordances (item selection and item relocation) as though they were desktop or touch-screen applications. Lastly, both games are essentially distraction-less. Students are only presented with a question and an answer set and there are no additional factors that might influence the students' performances, such as time pressure, audio or visual on-screen distractors. In the sections that follow, we describe game specifics and illustrate the students' MBEG interactions from an S2MR perspective by walking through an example problem from the Sea Formuli MBEG.

### 3.1. Suffizz: A Literacy Suffix Game Show

**Figure 1:**
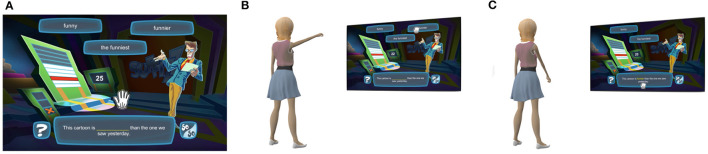
A student gesturing through a problem in the Suffizz Motion Based Educational Games (MBEG). **(A)** A student is presented with a multiple choice English question to solve using hand gestures and full body movement. **(B)** The student performs a mid-air hand gesture to select a word. **(C)** The student bends their body to move the selected word to the blank space and complete the sentence.

Suffizz is themed as a game show, in which students (e.g., game show contestants) practice their English grammar to further develop their literacy ability. The student is presented with an English sentence with a missing term, and 3 terms to select from (i.e., the potential answers, as shown in Figure 1A). To answers a question, the student must read the sentence and determine the correct answer from the given terms, perform a grabbing gesture to select their desired answer, and then move their selected answer to the blank space in the sentence located at the bottom of the screen. Once a question had been answered, the selected word turned green if correct and red, otherwise. Questions involved the use of correlative conjunctions, irregular plural nouns, verb tenses, and regular and intensive pronouns. Figure 1 shows an exemplar flow of gameplay where a student must select the correct suffix for the word *funny* provided in the sentence “this cartoon is ___ than the one we saw yesterday.” The student is provided with three potential answers: funny, the funniest, and funnier.

### 3.2. Sea Formuli: An Underwater Arithmetic Operations Game

**Figure 2:**
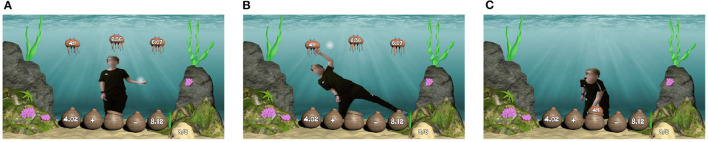
A student gesturing through a problem in the Sea Formuli MBEG. **(A)** A student is presented with a multiple choice maths problem to solve using hand gestures and full body movement. **(B)** The student performs a mid-air hand gesture to select an answer jellyfish. **(C)** The student bends their body to move the selected jellyfish to the empty basket to complete the equation.

Sea Formuli focuses on developing students' algebraic thinking through the practice of maths problems involving fractions, whole numbers, and decimals. Each question is an arithmetic equation relating to 3 terms, yet missing either an operator or operand. Questions are represented by a collection of baskets sitting on the ocean floor. Potential answers to choose from, presented as three jellyfish, floating at the top of the screen, are labeled with either an operand or an operator (as shown in Figure 2A). The student must determine the missing value which correctly completes the equation. To answer the question, the student must use a hand gesture (i.e., grabbing motion) to select the jellyfish containing their perceived correct answer (Figure 2B). Once a jellyfish is selected, the two non-selected jellyfish immediately float off screen. The student must move their body by bending down to place the jellyfish into the empty basket (as shown in Figure 2C). The operand (or operator) is then displayed on the basket, and the question is evaluated. The basket text turns green if correct and red otherwise.

### 3.3. S2MR Process in Sea Formula Breakdown

**Figure 3:**
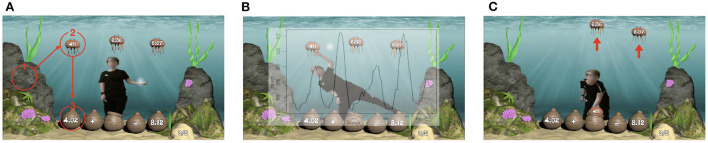
Description of detecting the three phases. **(A)** *See-Solve:* The first fixations on the question marks the beginning of the see-solve phase. **(B)** *Move:* The beginning of the peaks in the movement data mark the beginning of the move phase. **(C)** *Respond:* The moment of selection marks the beginning of the respond phase.

In this section, we describe the three phases of the S2MR cycle (Figure 3) exemplified by the Sea Formuli MBEG, by tracing through a student's interactions with a single question. Though this example is Sea Formuli specific, both games share the same multiple choice fill-in-the-blank question format (with questions at the base of the screen and answer options at the top of the screen) and utilize the same physical interaction mechanisms (e.g., a mid-air grabbing selection gestures). Thus, the following description can be generalized to explain how the S2MR cycle takes shape in the Suffizz MBEG as well.

Consider the addition question, 4.02+ _= 8.12, with potential answers: 4.1, 6.36, and 6.07 (Figure 3A). The *See-Solve* phase begins upon the student's first fixation on the question text (i.e., the baskets on bottom of the screen reading 4.02+ _= 8.12, Figure 3B), as detected *via* eye-tracking glasses. In this phase, a student must read, understand, and mentally solve the proposed question. The student may cycle between question and potential answers (i.e., comparing potential answers to each other or to the question), or they may only look at the answers one time, prior to determining their answer. The following *Move* phase (Figure 3C) begins as the student moves their body to initiate selecting their desired answer (the jellyfish labeled as 4.1) *via* mid-air grabbing hand gesture. The beginning of this phase (and end of the see-solve phase) is detected as the student's movement surpasses an individual threshold computed from the student's Kinect skeletal data (as shown in Figure 3B), and it lasts until the jellyfish has been selected. In the final *Respond* phase, the student moves their entire body to relocate their selected answer jellyfish to the empty basket, thereby completing the equation (as shown in Figure 3C). This phase begins the moment the answer jellyfish (labeled as 4.1) has been selected, as detected in the game logs.

## 4. Methods

### 4.1. Context

Our study took place in a local public elementary school and science museum in a European city. After receiving a thorough description of the study from school teachers and researchers (also the authors), students volunteered to participate on their own accord. In each location, the study was conducted by the researchers in a room strictly dedicated to the experiment set up to run two experimental setups in parallel.

### 4.2. Participants

Our sample includes 40 typically developing students (26 F, 14 M) with an average age of 10.9 years (*SD* = 1.09, *min* = 9, *max* = 12 years). In total, thirty students participated at the elementary school, and ten students at the science center. Students played 6 consecutive MBEG sessions (3 games of each game), totalling between 9 and 17 min in total. In exchange for their participation, students received a gift card. Prior to running the study, the national human research ethics organization. All students and their guardians were required to provide verbal/written informed assent/consent, respectively, prior to participation.

### 4.3. Procedure

We conducted an *in-situ* experiment that used wearables and physiological sensors to investigate the physio-cognitive states experienced by children as they interacted with two different MBEG. The students were given an Empatica E4 wristband (Emp, 2021) and pair of Tobii eye-tracking glasses (Olsen, 2012) to wear. The students played three consecutive games of Sea Formuli (see section 3.2) and three consecutive games of Suffizz (see section 3.1). Each game consisted of five algebraic questions. Students engaged in a practice session of each game and were given an opportunity to ask the experimenter questions, in order to ensure a proper understanding of the games' interaction mechanics prior to the beginning of game play. None of the children had prior exposure to MBEG. To reduce the novelty effect, each child was given 1–2 rounds of practice so that they get used to the learning environment.

### 4.4. Data Collections

We gathered wearable and physiological sensors data from three different sources: eye-tracking, wristband (with sensors for HRV, blood-pressure, temperature and EDA levels), and kinect skeleton data. We used data from all three sources to detect S2MR phases, and we used only eye-tracking and wristband data to compute MMD measurements. Prior to data collection, all the ethical permissions were obtained. It is important to point out here that neither eye-tracking data nor the data collected by Empatica E4 wristband could be used to trace individual children. Moreover, the children were given a code and there is no record of the code-name pairs. The data is kept on the secured servers of the university and a protected hard drive that is accessible to the authors only.

**Eye-tracking:** We collected students gaze data using Tobii eye-tracking glasses, with a sampling rate of 50 Hz and a one-point calibration. The students' field of view was captured using the Tobii glass controller software and an objective camera built into the nose bridge of the glasses. The video footage has a resolution of 1920 x 1080 at 25 Frames Per Second (FPS).

**Empatica E4 wristbands:** We collected four different variables from the students' wrist-data: EDA (64 Hz), HRV (1 Hz), skin temperature (4 Hz), and BVP (4 Hz). However, for the purpose of this study, we only used the first two variables.

**Kinect Skeleton:** Students' skeletal data was recorded at a sampling rate of 1Hz, using a Microsoft Kinect sensors. This data consisted of 3D position for the following 25 joints: head, shoulder-center, spine and hip-center, as well as hand, wrist, elbow, shoulder, feet, ankle, knee, and hip (both left and right for the last 8), as shown in the left image in **Figure 5**.

**Screen Recording Video:** We used Camtasia to record the screen that the children were interacting with for having a ground truth for processing eye-tracking data.

### 4.5. Data Pre-processing

**Figure 4:**
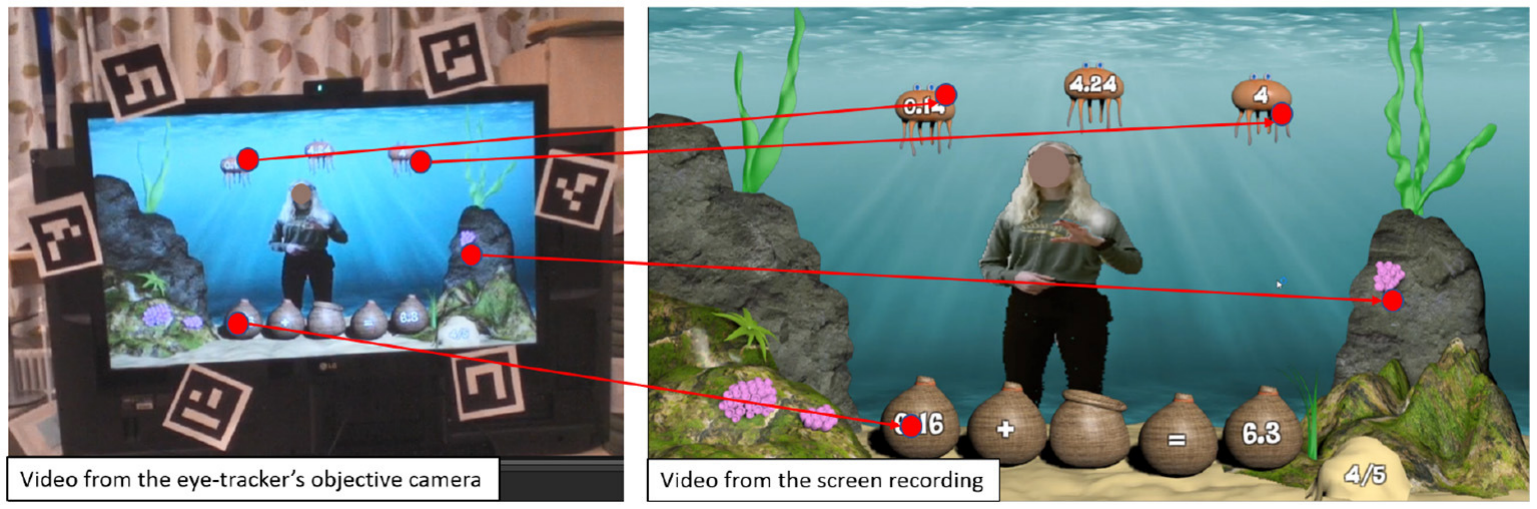
Example of homography calculation. The fiducial markers are the black and white tags fastened to the screen (*left*). The red dots, connected by a red line, represent corresponding locations in the different video recordings (ground truth and objective view from eye-tracking glasses). The correspondence matrix allows us to determine this line.

**Eye-tracking:** Fixations and saccades were identified using Tobii's default algorithm (for details refer to Olsen, 2012). A filter was applied to remove raw gaze points that were classified as blinks. Pupil dilation was normalized using the methods described in Lee-Cultura et al. (2021). Finally, in the final eye-tracking data pre-processing step, we computed the correspondences between the video from the eye-tracker's objective camera (**objective video**) and the screen recording video (**ground-truth**). These correspondences are called **homographies**. This process was adopted from Lee-Cultura et al. (2021) (the process is shown in the Figure 4).

**Wrist band:** A simple smoothing function was applied to the time series of the EDA and HR (to remove unwanted spikes). We divided our signal in to windows, where each ‘window' described a time segment containing 10 s of data, and successive windows contained a 5 s overlap. Similar to pupil dilation, EDA and HR are highly susceptible to personal and contextual biases; such as pre-existing physical health conditions, time of the day, the students's age, gender, and amount of sleep. To remove the subjective and contextual bias from the data, we normalized EDA and HR using the first 30 s of the data streams.

**Kinect Skeleton:** No pre-processing was required.

### 4.6. Multimodal Measurements

In this section, we define the MMD measurements used in this paper. All the data was aggregated using a 10 s window throughout the different phases of the interaction. Once the windows were formed, all the measurements were computed and normalized between zero and one using a MinMax normalization process. Once the data were normalized, we used measurements from relevant literature to capture the student's learning. The selected measurements have been shown to have distinguished ability across different performance levels. Table 1 provides the definition and appropriate reference for each measurement. After computing measurements from the MMD, we also computed the features associated with each measurement, as shown in Table 1. These features were selected based on their previously demonstrated high predictive power regarding performance prediction Sharma et al. (2019b, 2020c) and have been used in contemporary multimodal research for education and problem-solving (Blikstein and Worsley, 2016; Andrade et al., 2017; Worsley and Blikstein, 2018; Lee-Cultura et al., 2020b).

**Table 1:** Definitions of the features computed from the MMD measurements described in section 4.6.

**Feature type**	**Definition**
Value histogram	Mean, median, SD, skewness, kurtosis of the values.
ARMA	Auto-regressive moving average: maps the current value to the history of time series.
GARCH	Generalized Auto-regressive conditional heteroscedasticity: maps the current variance to the historical variance of time series and the heterogeneity of the appearance of the values.

**Cognitive load** is a gaze-based proxy to the mental effort invested when solving a given problem (Palinko et al., 2010; Joseph and Murugesh, 2020). We used eye-tracking data to compute cognitive load as a function of pupillary activity (Duchowski et al., 2018).

**Figure 5:**
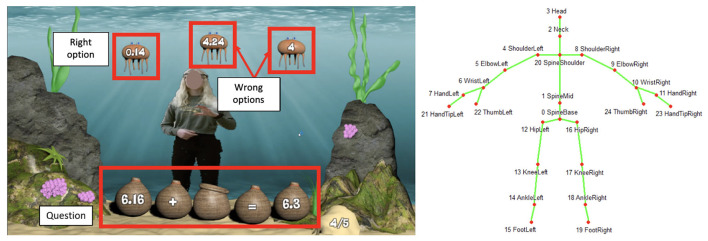
Left: example AOIs for a question. We defined three AOIs on the screen, (1) the question, (2) the right option, and (3) the wrong options. Shows an example of the AOI positions for a single question. The question always remained at the same position on the screen but right and wrong options changed their positions for the different questions randomly. Right: Schematic representation of the joints as detected by the Kinect sensor.

**Transitions between Areas of Interest (AOIs)** are indicative of “how learners are processing the visual information.” This measurement is mostly used in multimedia learning scenarios (Ginns, 2005; Sung and Mayer, 2012; Khacharem et al., 2013) to examine whether the learners are processing the information in a manner that improves the learning performance. After the AOIs were defined on the stimulus screen (Figure 5 left), we computed the percentage of three types of gaze transitions between distinct AOIs: between question and right option; between question and wrong options; and between right and wrong options.

**Information Processing Index** is the ratio between global and local information processing. Global processing is a series of short fixations and long saccades, while local processing is a series of long fixations and short saccades. A high value of this index indicates a large area of screen explored per unit time. This index shows how much information is received by the learners in a given period of time (Unema et al., 2005; Poole and Ball, 2006).

**Saccade Velocity**: is the velocity of the saccades and is related to the perceived difficulty of a problem-solving task (Bronstein and Kennard, 1987; Smit and Van Gisbergen, 1989).

**Mean HR**: corresponds to the mean HR of the child per second. An increase in HR is often related to stressful situations (Harada, 2002; Herborn et al., 2015).

**Number of EDA peaks** is computed using the method proposed by Di Lascio et al. (2018) and is often associated with physiological arousal (Di Lascio et al., 2018; Gashi et al., 2019) and engagement (Hernandez et al., 2014).

**Phasic EDA level**: EDA signal is comprised of two parts: the tonic and phasic components. The tonic component of the EDA signal is the one with slow evolving patterns. The phasic component of the EDA signal is the one with rapid changes and is found to be related to physiological arousal (Di Lascio et al., 2018). In this paper, we consider only the mean phasic EDA component as a measure of physiological arousal (Hasson et al., 2008; Leiner et al., 2012).

### 4.7. Data Analysis

To address our first RQ (determining the association between students' MMD measurements/features and their learning performance during different phases of the S2MR cycle), we used two separate methods: inferential statistics using the MMD measurements and predictive modeling with MMD features. To answer our second RQ, (informing the design of a physio-cognitive aware agent using MMD), we present results based on the novel CFE. In the sections that follow, we present details concerning inferential statistics and predictive modeling, and then we introduce the CFE and related terms.

#### 4.7.1. Inferential Statistics

The measurements described in section 4.6 were normalized using a **MinMax** normalization (with the exception of time to the first fixation on the question). Each variable was computed for all three phases of the interaction, as defined by the S2MR cycle, namely, See-Solve, Move, and Respond. We used a **repeated-measure ANOVA** to test the differences between these measurements and the correctness of each answer provided by students. **The student's unique ID was used as the grouping variable**. Prior to this, we also checked for normal distribution (using a **Shapiro-Wilk Test**) and homoscedasticity of the measurements (using a **Breush-Pagan test**). We used **z-transforms** to normalize the distribution (e.g., cognitive load and mean HR) when the variables were not normally distributed. If the variables were not homoscedastic, we used a **Welch correction** for the ANOVA (e.g., saccade velocity, Information Processing Index (IPI), amount of movement, posture stability, heart rate, phasic EDA, number of EDA peaks). Further, a Bonferroni correction was applied to counteract the effect of multiple comparisons on the *p*-values of the tests.

#### 4.7.2. Predictive Modeling

In machine learning, ensemble models combine the decisions from multiple models to improve overall prediction accuracy. They have been shown to be advantageous over individual predictive models (Avnimelech and Intrator, 1999; Gavrishchaka et al., 2010; Qiu et al., 2014). In this paper, we combine predictions from 7 different algorithms: Support Vector Machines (Chapelle and Vapnik, 2000) with linear, radial, and polynomial kernels; Gaussian process models (Williams and Rasmussen, 2006) with linear, radial, and polynomial kernels; and M5 model trees. These methods are designed to improve the stability and accuracy of machine learning algorithms. One way of using the results from multiple models is to use a weighted average from all of the prediction algorithms. The weights for individual predictions are determined according to their accuracy during the validation phase. There are 3 major advantages of these methods (Avnimelech and Intrator, 1999; Gavrishchaka et al., 2010; Qiu et al., 2014):

1. We can compare the performance of the ensemble methods to the diversification of our models predicting cognitive performance. It is advised to keep a diverse set of models to reduce the variability in the prediction and hence, to minimize the error rate. Similarly, the ensemble of models yields a better performance on the test case scenarios (unseen data), as compared to the individual models in most cases.
2. The aggregate result of multiple models involves less noise than the individual models. This leads to model stability and robustness.
3. Ensemble models can be used to capture the linear and non-linear relationships in the data. This can be accomplished by using two different models and forming an ensemble of the two.

We performed **out-of-sampling testing** (i.e., leave-one-participant-out), dividing the data-set into 3 subsets: 1) training, 2) validation, and 3) testing set. The data set was split based on student identifiers. The testing set was put aside (10 % based on student ID). All of the models were trained and validated using the training and validation sets with cross-validation. The cross-validation was also performed using leave-one-participant-out. We observed our data set to be heavily unbalanced. Particularly, it contained five times more right answers than wrong answers. To account for this, we applied Synthesizing Minority Oversampling Technique (SMOTE) Lusa et al. (2013). We implemented the SMOTE strategy by identifying the **five nearest neighbors for each original point of the minority class** and then added four new (synthetic) points. The **five new points were generated** using the mean of the original point's four closest neighbors and then adding/subtracting 25 and 50%, respectively, of the SD of the four neighbors to/from the mean.

The following metrics were used to evaluate the performance of the ensemble classifier:

**Precision** = TP / (TP + FP);

**Recall** = TP / (TP + FN);

**F1 score** = 2TP / (2TP + FP + FN).

Where, **TP** = true positive; **FP** = false positive; **TN** = true negative; **FN** = false negative. For the purpose of evaluating prediction quality, the “right” class is the “positive” class. For the baseline prediction, we selected the “majority class baseline,” rather than the “random allocation baseline,” to accommodate for the skewed nature of our data-set.

#### 4.7.3. Carry Forward Effect

In order to understand the CFE, we first introduce the concept of “affinity” as the “direction of relation.” In a *t*-test (or ANOVA or any other between group comparison), we regard affinity as the direction of the higher value. For example, if the students' attention is higher for the right answers (than for wrong answers), we say that “attention has affinity with the right answer.” If students' stress is higher for wrong answers (than for right answers), then we say that “stress has affinity with the wrong answer.” For correlations tests (parametric and non-parametric), the affinity matches the sign of the correlation coefficient (either negative or positive).

We define CFE from three perspectives: (1) inferential statistics; (2) predictive modeling; and (3) design of an artificially intelligent agent that provides feedback to support learning.

**Figure 6:**
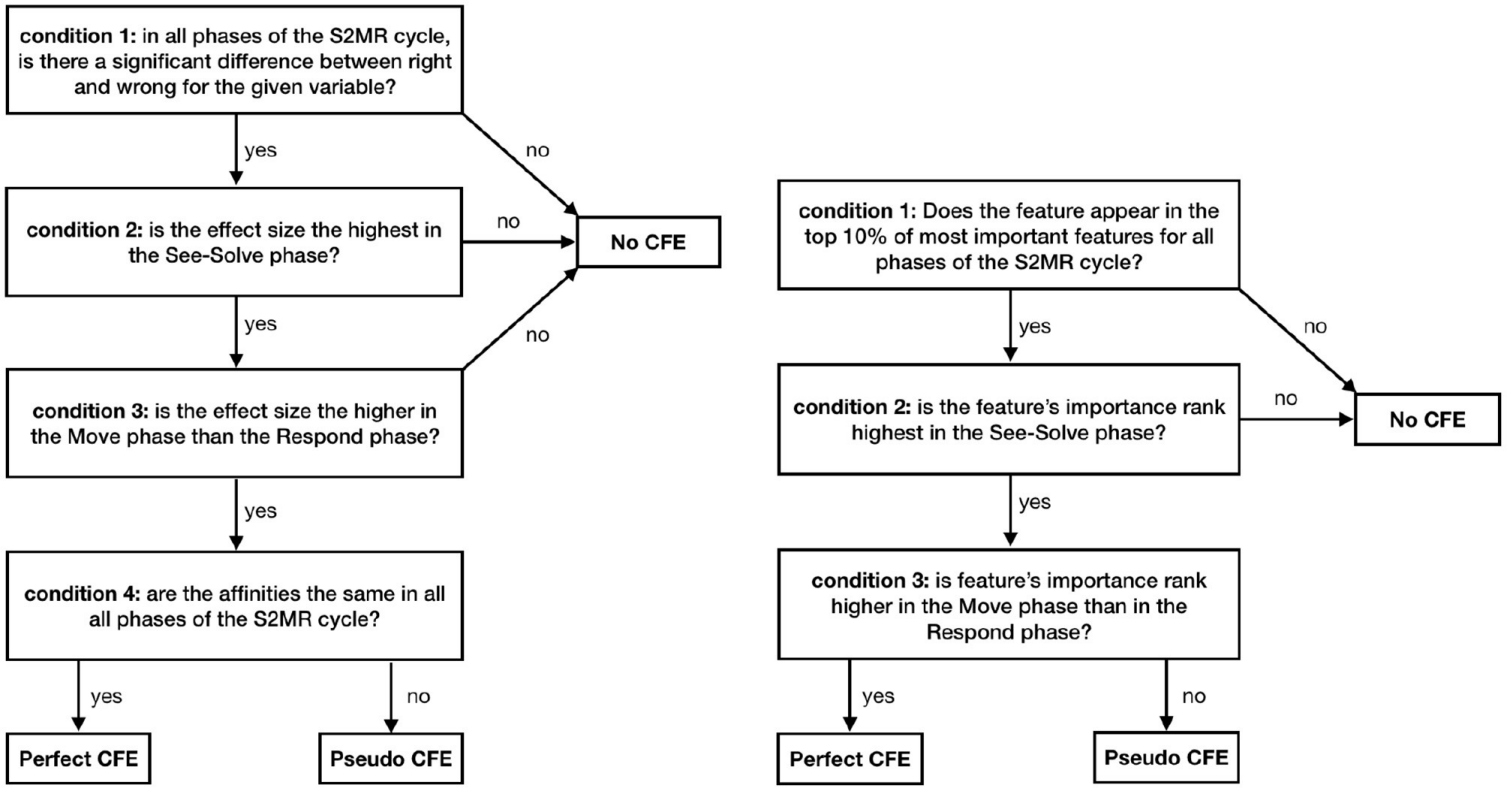
A classification scheme for the three classes of Carry Forward Effect (CFE) by inferential statistics (left) and predictive modeling (right).

From the *inferential statistics* perspective, CFE is defined by the following 4 conditions (Figure 6, left): (1) in all three phases of the S2MR cycle, there is a significant difference for a given variable between the correctness levels (i.e., right or wrong); (2) the significant difference has the highest effect size in the See-Solve phase; (3) the effect size is higher in the Move phase than the Respond phase; and (4) all affinities are in the same direction. Moreover, we define three classes of CFE according to these conditions. Given that all four conditions hold true, we have *Perfect* *CFE*. If all conditions are true, except for condition four, we have *PseudoCFE*. Otherwise, there we have no CFE.

From the predictive modeling perspective, CFE is defined by the following 3 conditions (Figure 6, right): (1) in all three phases of the S2MR cycle, the given feature appears in the top 10% of the most important features; (2) according to feature importance, the feature's rank^2^ is the highest in the See-Solve phase; and (3) the feature's rank is higher in the Move phase than in the Respond phase. We justify limiting the *most important* features to the top 10% to keep the discussion of CFE related measurements to a reasonable number. In practice, this limit can be set to any scaled variable importance in the predictive model (e.g., 0–1 or 0-100) or any top percentage (e.g., *x*%). The choice belongs to the researchers, designers, and practitioners, and depends on the number of measurements desired for future consideration (e.g., as metrics to be integrated into AI agent). Furthermore, we then define three classes of CFE (i.e., Perfect, Pseudo, and None) according to these conditions as follows. When all three conditions are true, we have Perfect CFE. If all conditions are true, except for condition three, we have Pseudo CFE. Otherwise, we have no CFE. The role of a predictive feature (e.g., used to predict learning performance) is to explain the variance in the learning performance variable. This predictive feature might or might not have the same predictive power in the various phases. Moreover, it can or cannot be in the list of most important features for the prediction. A measurement/feature showing CFE has to be in the first quartile of the feature importance and rank should be decreasing from the problem-solving phase to a non-problem-solving-phase.

## 5. Results

Concerning the correctness of the answer (right or wrong), there were no significant differences between the three phases of the S2MR cycle (i.e., See-Solve, Move, and Respond). Additionally, we did not find gender or age bias connected to the correctness of students' responses. This section is organized as follows: (1) we present inferential statistics results from the individual data streams (i.e., eye-tracking, physiological, and motion); (2) we discuss the CFE results using inferential statistics; (3) we present the predictive modeling results derived from combining the MMD; (4) we present the CFE results using predictive modeling. The first and third parts answer the **RQ1** (association of MMD measurements with the learning performance). Whereas, the second and fourth parts provide address **RQ2** (the design of a physio-cognitive aware agent).

### 5.1. Sea Formuli Eye-Tracking Results

**Figure 7:**
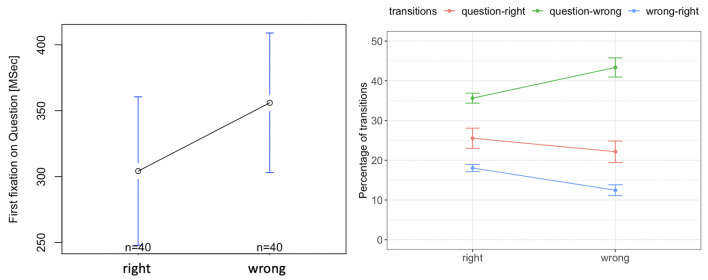
Tobii eye tracking results for the Sea Formuli MBEG. Left: time to the first fixation on the question. Right: percentage of transitions between the question, right and wrong options.

We observed no significant difference between the time to the first fixation on the question from right and wrong options [*F*_(1, 39)_ = 1.83, *p* = 0.17, Figure 7, left]. There was also no significant difference in the percentage of transitions between the question and the right option [*F*_(1, 39)_ = 3.02, *p* = 0.09, red curve in Figure 7, right]. However, the percentage of transitions between the wrong options and question was significantly higher for the wrong responses, than for the right responses [*F*_(1, 39)_ = 29.20, *p* = 0.00001, green curve in Figure 7, right]. Further, the percentage of transitions between the wrong options and the right option was higher and was significantly higher for the right response than for the wrong response [F_(1, 39)_ = 41.59, *p* = 0.000001, blue curve in Figure 7, right]. This indicates that for the right responses, students compare all options more than when they provide a wrong response.

**Figure 8:**
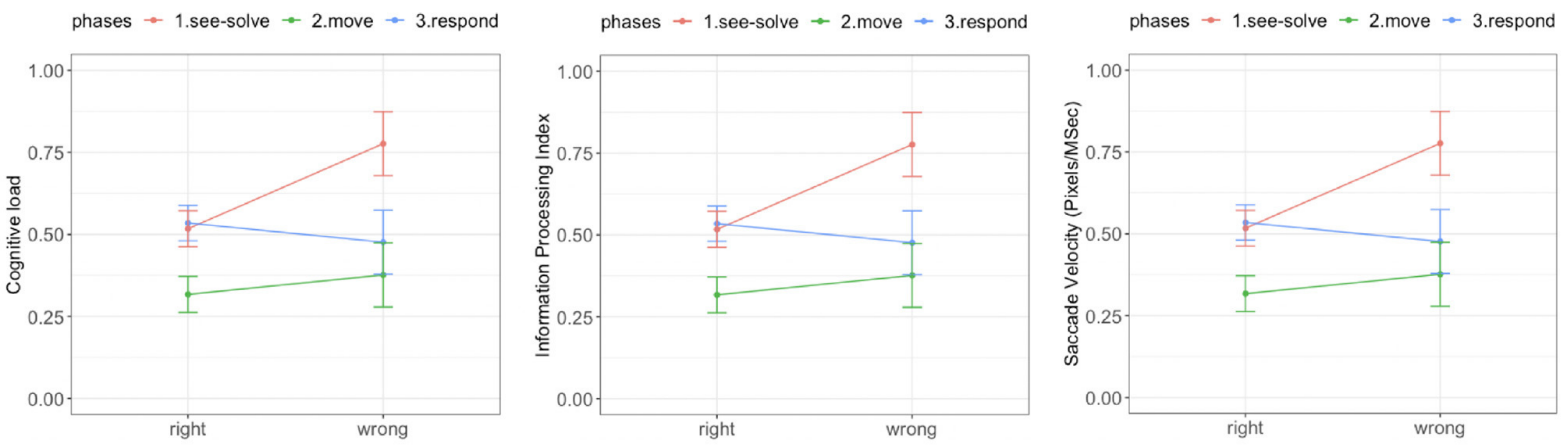
Tobii eye tracking results for the Sea Formuli MBEG. Left: Cognitive load. Middle: Information processing index. Right: Saccade velocity.

**There was a significant difference in cognitive load during the See-Solve phase associated with the correctness of answer** [*F*_(1, 39)_ = 19.34, *p* = 0.00001, red curve in Figure 8, left]. **The cognitive load associated with wrong responses was significantly higher than with right responses during the See-Solve phase** (Figure 8, left). However, there was no difference in cognitive load associated with the correctness of the answers during either the Move [*F*_(1, 39)_ = 1.01, *p* = 0.31, green curve in Figure 8, left] or Respond [*F*_(1, 39)_ = 0.98, *p* = 0.32, blue curve in Figure 8, left] phases.

There was a significant difference in the IPI associated with the correctness of the answers during all three phases (Figure 8, middle). **The** **IPI** **associated with right answers was significantly lower in all three phases than the** **IPI** **associated with wrong answers**. However, this difference reduced as students transitioned from the See-Solve phase [*F*_(1,37.04)_ = 14.25, *p* = 0.0003] to the Move phase [*F*_(1,30.56)_ = 7.29, *p* = 0.008], to the Respond phase [*F*_(1,26.21)_ = 4.14, *p* = 0.04].

Similarly, we observed significant differences in saccade velocity associated with the correctness of the answers during all three phases of the S2MR cycle (Figure 8, right). **The saccade velocity associated with right responses were significantly lower for all three phases than the saccade velocity associated with the wrong responses**. However, the differences reduced from the See-Solve phase [*F*_(1,36.06)_ = 15.24, *p* = 0.0003] to the Move phase [*F*_(1,37.18)_ = 3.95, *p* = 0.05], but then *increased* as students transitioned to the Respond phase [*F*_(1,25.31)_ = 11.42, *p* = 0.001].

### 5.2. Sea Formuli Empatica E4 Results

**Figure 9:**
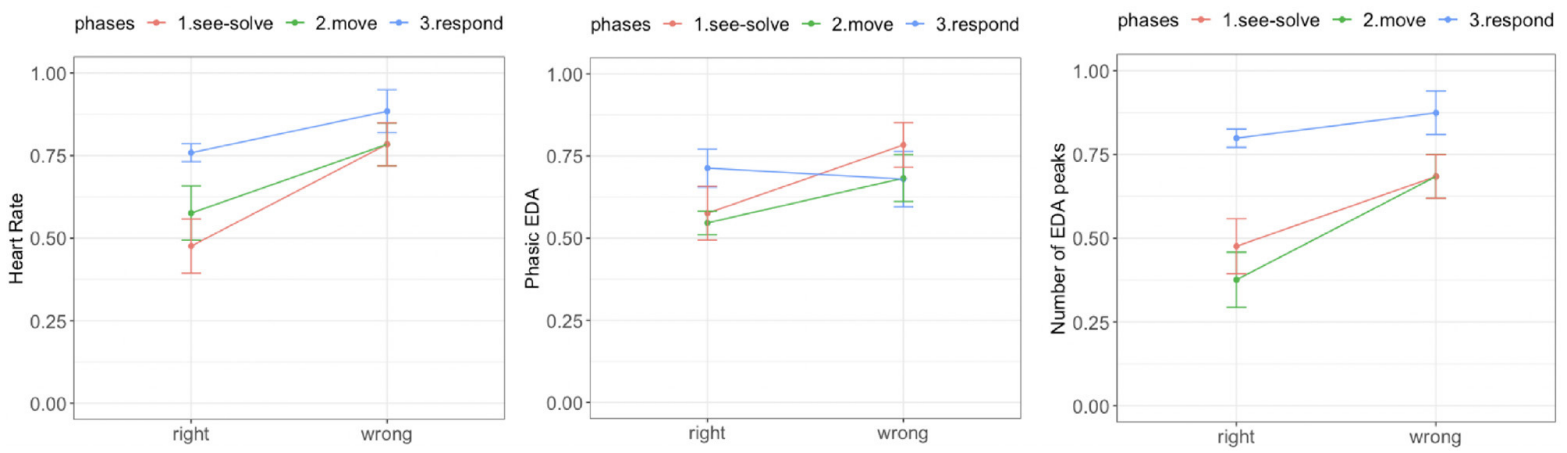
Empatica E4 results for the Sea Formuli MBEG. Left: Mean heart rate. Middle: Phasic electrodermal activity (EDA). Right: Number of EDA peaks.

We observed a significant difference in mean HR between the right and wrong responses. During each phase of the S2MR cycle, the wrong response was associated with a higher mean HR (Figure 9, left). We also observed a diminishing difference as students transitioned from See-Solve [*F*_(1,37.65)_ = 31.21, *p* = 0.00001] to Move [*F*_(1,37.65)_ = 14.25, *p* = 0.0003], to Respond phase [*F*_(1,26.45)_ = 11.42, *p* = 0.001].

We did not observe a difference in tonic EDA levels for the right and wrong responses during any phase of the S2MR cycle [solve: *F*_(1, 39)_ = 3.11, *p* = 0.08; move: *F*_(1, 39)_ = 2.44, *p* = 0.12; answer: *F*_(1, 39)_ = 1.01, *p* = 0.31]. Further, there was no significant difference between phasic EDA levels for the right and wrong response during the Respond phase [*F*_(1,34.37)_ = 0.38, *p* = 0.53]. However, phasic EDA levels were higher for the wrong responses than for the right response during both the See-Solve [*F*_(1,37.87)_ = 13.61, *p* = 0.0004] and Move [*F*_(1,27.51)_ = 10.51, *p* = 0.002] phase (Figure 9, middle).

Finally, for all three phases of the S2MR cycle, a wrong response was associated with a higher number of EDA peaks (Figure 9, left). However, this difference is highest during the Move phase [*F*_(1,37.04)_ = 31.21, *p* = 0.00001], followed by the See-Solve [*F*_(1,31.78)_ = 14.25, *p* = 0.0003], and lastly, the Respond phases [*F*_(1,27.21)_ = 4.14, *p* = 0.05].

### 5.3. Sea Formuli CFEE: Inferential Statistics

Table 2 shows the effect sizes from the ANOVA (with or without Welch correction) for the different measurements across correctness of answers (i.e., right and wrong) during the phases of the S2MR cycle. For IPI and mean HR, the effect sizes *decrease* from the See-Solve to Move to Respond phase. Further, the effect sizes associated with these transitions (See-Solve to Move, and Move to Respond) have the same affinities between the given measurement and dependent variable (e.g., both mean HR and IPI are always lower for the correct response). **Thus, we conclude that IPI and mean HR demonstrate a Perfect CFE**.

**Table 2:** The effect sizes for the MMD measurements for the three phases and the corresponding CFE they exhibit.

	**Sea Formuli effect size**				**Suffizz effect size**			
**Measurement**	**See-Solve**	**Move**	**Respond**	**CFE Type**	**See-Solve**	**Move**	**Respond**	**CFE Type**
Cognitive load	0.26	0.06	0.06	none	0.45	0.29	0.05	none
Information processing index	0.22	0.16	0.12	Perfect	0.32	0.18	0.12	Perfect
Saccade velocity	0.21	0.12	0.17	Pseudo	0.21	0.15	0.12	Perfect
Mean Heart Rate	0.31	0.21	0.16	Perfect	0.41	0.21	0.17	Perfect
#EDA peaks	0.21	0.31	0.21	none	0.39	0.32	0.13	Perfect
Tonic EDA	0.09	0.11	0.04	none	0.03	0.10	0.03	none
Phasic EDA	0.21	0.18	0.01	none	0.34	0.21	0.01	none

Considering saccade velocity, the effect size for the See-Solve phase is the highest, followed by the Response phase, and then the Move phase. Moreover, all effect sizes have the same affinity between the saccade velocity and the correctness of the answers (e.g., always lower for the right response). **Therefore, the saccade velocity exhibits Pseudo CFE**.

The difference in cognitive load between right and wrong responses was only significant during the See-Solve phase indicating no CFE. The number of EDA peaks was also significantly different between the right and wrong responses for all three S2MR phases. However, the effect size for the See-Solve phase was not the highest. **Thus, we conclude that the number of EDA peaks does not display CFE**. Finally, concerning tonic EDA, there was no difference between the right and wrong responses, consequently, **we conclude that tonic EDA does not indicate CFE**.

### 5.4. Sea Formuli Prediction Results

Table 3 shows the prediction results for the correctness of answers. The random baseline for the prediction is low (precision = 0.50; recall = 0.50; F1-score = 0.50), while the majority class prediction baseline is very high (precision = 0.83; recall = 1.00; F1-score = 0.90). Thus, it is not possible to improve the prediction's recall. We note that by using data from the See-Solve phase, it is possible to improve the precision (0.89) and F1-score (0.91) by small margins. On the other hand, using data from the other two phases (i.e., Move and Respond), prediction recall cannot be improved. However, the precision of the performance prediction has improved in the last two phases when compared to the majority class baseline.

**Table 3:** Predictive modeling results for the correctness of the responses using the data from the three different phases.

	**Sea Formuli prediction results**			**Suffizz prediction results**		
**Phase**	**Precision**	**Recall**	**F1-score**	**Precision**	**Recall**	**F1-score**
See-Solve	0.8889	0.9333	0.9106	0.8913	0.9111	0.9011
Move	0.8730	0.9166	0.8943	0.8478	0.8667	0.8571
Respond	0.8438	0.9000	0.8710	0.8000	0.8511	0.8247

### 5.5. Sea Formuli CFE: Predictive Modeling

**Figure 10:**
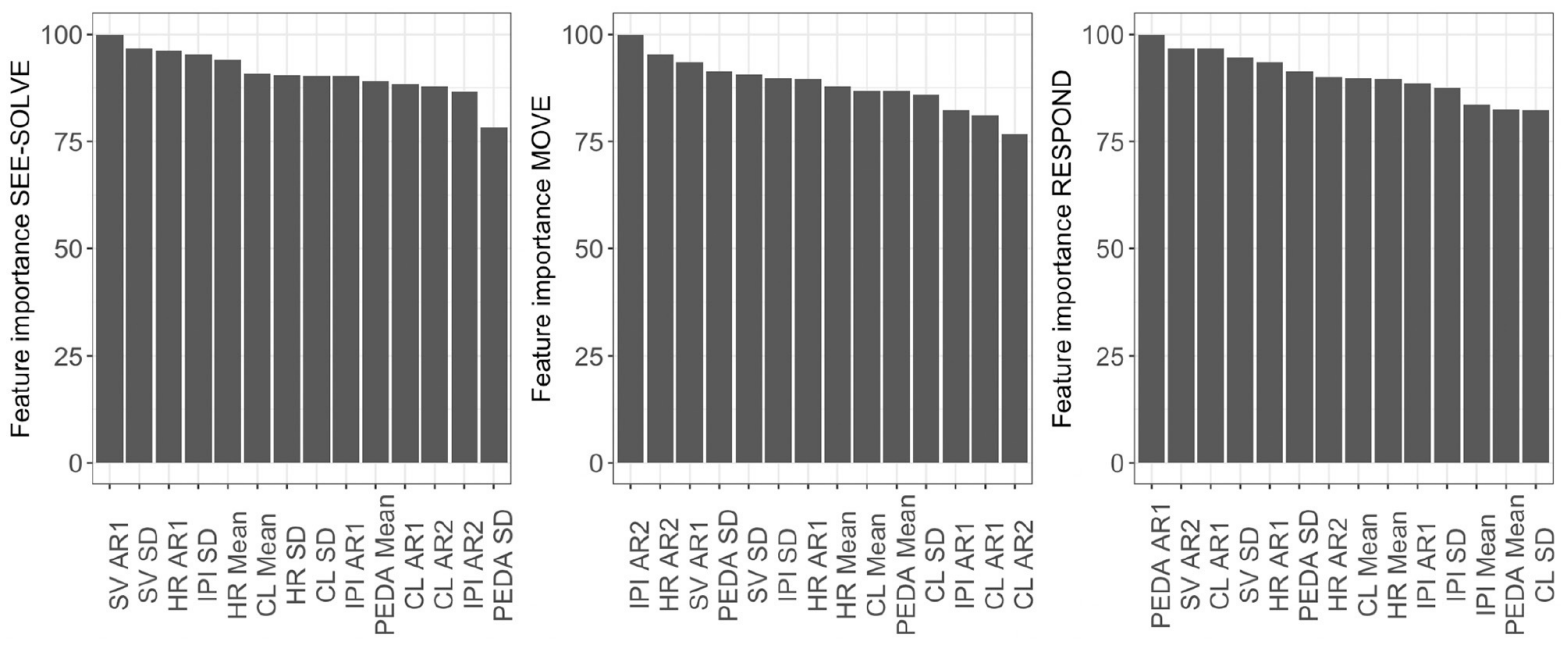
Variable Importance for predictive modeling using data from the different phases in Sea Formuli.

Considering the CFE from the predictive modeling, we note that mean cognitive load, mean HR, mean IPI, and IPI's first auto-regressive coefficient demonstrate **Perfect CFE** because their individual feature importance ranks are the highest in the See-Solve, followed by Move, then Respond phases (Table 4, Figure 10). The set of features with a **Pseudo CFE** includes: first AR coefficient for HR, second AR coefficient for cognitive loads, mean phasic EDA, and saccade velocity SD. This is because these features have their highest individual feature importance rank in the See-Solve phase, but their individual feature rank in the Move phase is smaller than in the Respond phase (Table 4). The remaining features do not exhibit any CFE for either of the following two reasons: (1) they do not appear in the top 10% most important feature list for any phase of the S2MR cycle, or (2) their highest individual rank does not occur in the See-Solve phase.

**Table 4:** Top 10% features from all the three phases, the ranks of these features in the three different phases and accordingly, the CFE they exhibit.

**Sea Formuli ranks for predicting correctness of an answer**					**Suffizz ranks for predicting correctness of an answer**				
**Feature**	**See-solve**	**Move**	**Respond**	**CFE Type**	**Feature**	**See-solve**	**Move**	**Respond**	**CFE Type**
HR Mean	5	6	8	Perfect	HR Mean	3	4	8	Perfect
HR SD	7	-	-	none	HR SD	13	-	-	none
HR AR1	3	7	5	Pseudo	HR AR1	14	9	2	none
HR AR2	-	2	7	none	HR AR2	-	2	-	none
PEDA mean	10	10	13	Pseudo	Peaks mean	11	14	11	Pseudo
PEDA SD	14	4	6	none	Peaks SD	8	3	14	none
PEDA AR1	-	-	1	none	Peaks AR1	-	-	4	none
CL Mean	4	8	9	Perfect	CL Mean	10	11	12	Perfect
CL SD	8	11	-	none	CL SD	9	13	3	none
CL AR1	11	13	3	none	CL AR1	2	10	6	Pseudo
CL AR2	12	14	14	Pseudo	CL AR2	7	1	5	none
SV SD	2	5	4	Pseudo	SV SD	1	5	1	Pseudo
SV AR1	1	3	-	none	SV AR1	12	7	-	none
SV AR2	-	-	2	none	SV AR2	-	-	10	none
IPI Mean	-	-	12	none	IPI Mean	-	-	7	none
IPI SD	6	9	11	Perfect	IPI SD	6	8	9	Perfect
IPI AR1	9	12	10	Perfect	IPI AR1	5	12	13	Perfect
IPI AR2	13	1	-	none	IPI AR2	4	6	-	none

### 5.6. Suffizz Eye-Tracking Results

**Figure 11:**
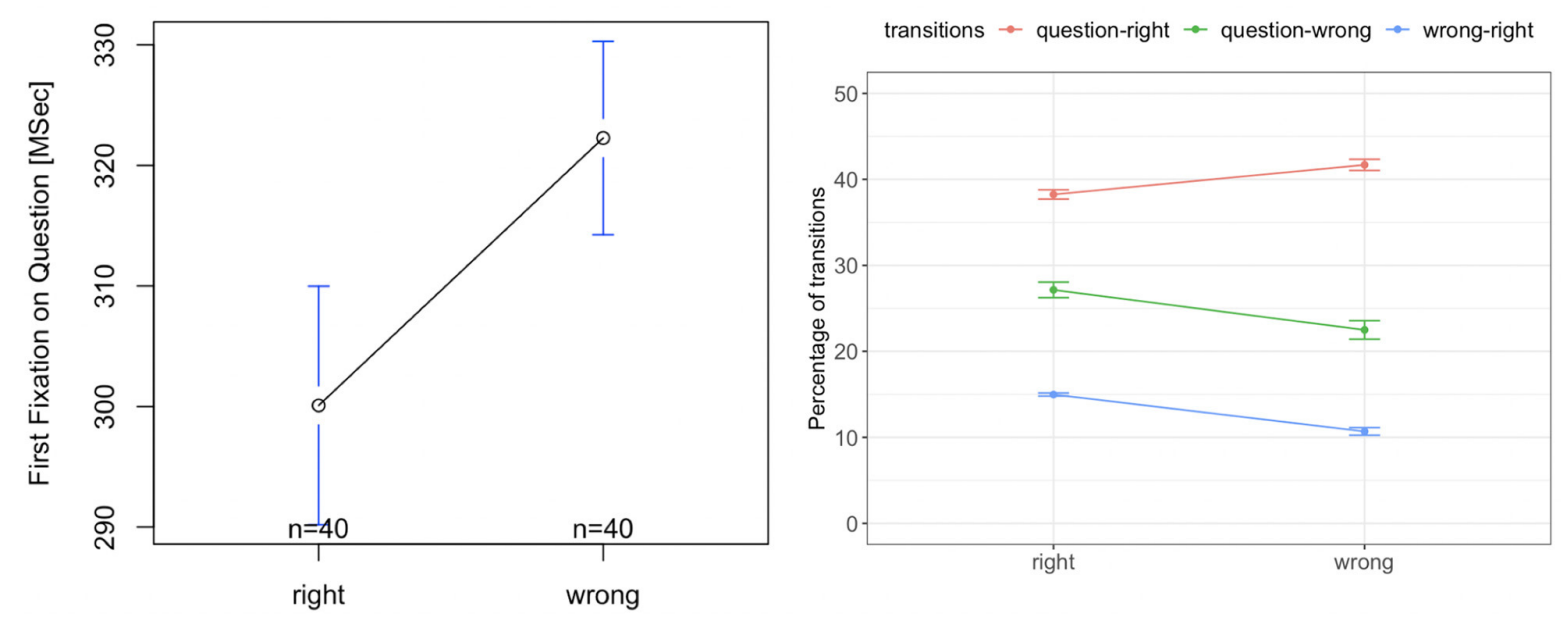
Tobii eye tracking results for the Suffizz MBEG. Left: time to the first fixation on the question. Right: percentage of transitions between the question, right and wrong options.

There was no significant difference in the time to first fixation on the question for the right or wrong responses [*F*_(1, 39)_ = 0.49, *p* = 0.32, Figure 11, left]. There was also no significant difference in the percentage of the transitions from the question to the right option [*F*_(1, 39)_ = 0.02, *p* = 0.91, Figure 11, right]. However, the percentage of transitions between the wrong options and question was significantly higher for the wrong response than that for the right response [*F*_(1, 39)_ = 19.33, *p* = 0.001, Figure 11, right]. Further, the percentage of transitions between the wrong options and the right option was significantly higher for the right response than for the wrong response [*F*_(1, 39)_ = 37.62, *p* = 0.0001, Figure 11, right]. This indicates that for right responses, students compare the options more than when they provide the wrong responses.

**Figure 12:**
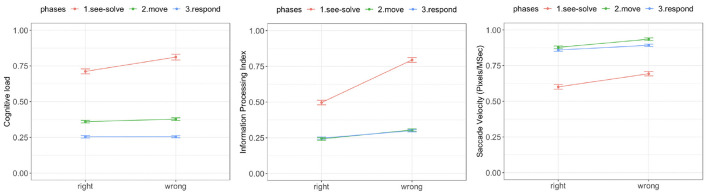
Tobii eye tracking results for the Suffizz MBEG. Left: Cognitive load. Middle: Information processing index. Right: Saccade velocity.

**There was a significant difference in cognitive load (**Figure 12**, left) associated with the correctness of answers during the See-Solve and Move phases** [See-Solve: *F*_(1, 39)_ = 25.97, *p* = 0.0001; Move: *F*_(1, 39)_ = 6.65, *p* = 0.01]. However, no such difference was detected during the Respond phase [*F*_(1, 39)_ = 0.05, *p* = 0.94]. **The cognitive load associated with wrong answers was significantly higher than with right answers during the See-Solve and Move phases**.

There was a significant difference in IPI (Figure 12, middle) associated with the correctness of the answers during all phases of the S2MR cycle. **The IPI associated with right responses was significantly lower in all three phases than the IPI associated with wrong responses**. However, this difference reduced as students transitioned from the See-Solve phase [*F*_(1,36.24)_ = 34.69, *p* = 0.0001] to the Move phase [*F*_(1,35.46)_ = 27.39, *p* = 0.0001], and finally to the Respond phase [*F*_(1,36.21)_ = 23.54, *p* = 0.0001].

Lastly, **the saccade velocity (****Figure 12****, right) associated with right responses was significantly lower during all three phases of the** **S2MR** **than the saccade velocity associated with the wrong responses**. However, there difference reduced from the See-Solve phase [*F*_(1,33.56)_ = 28.97, *p* = 0.0001] to the Move phase [*F*_(1,33.18)_ = 24.25, *p* = 0.0001] and then an *increase* in the difference as students transitioned to the Respond phase [*F*_(1,35.41)_ = 13.42, *p* = 0.001].

### 5.7. Suffizz Empatica E4 Results

**Figure 13:**
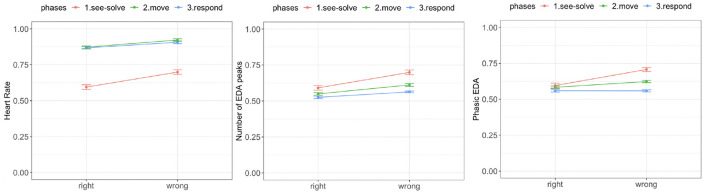
Empatica E4 results for the Suffizz MBEG. Left: Mean heart rate. Middle: Phasic EDA. Right: Number of EDA peaks.

We observed a significant difference in mean HR (Figure 13, right) between the right and wrong responses. For all three S2MR phases, the wrong response was associated with the higher mean HR. Additionally, the difference diminished as the students transitioned from the See-Solve [*F*_(1,36.54)_ = 38.32, *p* = 0.0001] to Move [*F*_(1,35.43)_ = 29.85, *p* = 0.0001], and finally to the Respond phase [*F*_(1,29.58)_ = 15.41, *p* = 0.001].

We observed no significant differences in tonic EDA levels for the right and wrong responses in any of phase of the S2MR cycle [See-Solve: *F*_(1, 39)_ = 1.81, *p* = 0.07; Move: *F*_(1, 39)_ = 1.56, *p* = 0.11; Response: *F*_(1, 39)_ = 0.98, *p* = 0.35]. Further, there was no significant difference between the phasic EDA levels for the right and wrong response during the Respond phase [*F*_(1,33.26)_ = 0.01, *p* = 0.89]. However, phasic EDA (Figure 13, right) levels were higher for the wrong responses than for right responses during both the See-Solve [*F*_(1,34.53)_ = 28.47, *p* = 0.0001] and Move [*F*_(1,37.62)_ = 21.23, *p* = 0.0001] phases.

Lastly, in all three phases of the S2MR cycle, a wrong response was associated with a higher number of EDA peaks (Figure 13, middle). This difference was the highest for the See-Solve phase [*F*_(1,35.84)_ = 38.47, *p* = 0.0001], followed by the Move [*F*_(1,35.23)_ = 34.37, *p* = 0.0001], and finally, the Respond phase [F_(1,24.27)_ = 24.34, *p* = 0.0001].

### 5.8. Suffizz CFE Results–Inferential Statistics

Table 2 shows the effect sizes from the ANOVA (with or without Welch correction) for the different measurements across the correctness of answers (right and wrong) during the three phases of the S2MR cycle. For IPI, saccade velocity, number of EDA peaks, and mean HR, the effect sizes *decreased* from the See-Solve to the Move to the Respond phase. Further, the effect sizes associated with the phase transitions (See-Solve to Move, and Move to Respond) had the same affinity for a given measurement and dependent variable (e.g., all four measurements were lower for the correct response). **Thus, we conclude that IPI, saccade velocity, number of EDA peaks, and mean HR demonstrate a Perfect CFE**. Moreover, the difference in cognitive load and phasic EDA, between right and wrong response, was only significant during the See-Solve and Move phases. **Therefore, cognitive load and phasic EDA do not show CFE**. Finally, **we conclude that tonic EDA does not indicate CFE**, since there was no difference between the right and wrong responses for tonic EDA levels.

### 5.9. Suffizz's Predictive Modeling Results

Table 3 shows the prediction results for the correctness of answers. The random baseline for the prediction is low (precision = 0.50; recall = 0.50; F1-score = 0.50), while the majority class prediction baseline is very high (precision = 0.75; recall = 1.00; F1-score = 0.85). This indicated that it is not possible to improve the prediction's recall. However, we can improve the precision (0.89) and F1-score (0.90) by considerable margins by using the data from the See-Solve phase. In the Move phase, precision (0.85) can also be improved considerably but only marginal improvements are able for the F1-score (0.86, rounded up to two digits). Finally, using data from the Respond phase, it is possible to improve the precision (0.80) considerably, but it is not possible for either of the other metrics.

### 5.10. Suffizz's Carry Forward Results–Predictive Modeling

**Figure 14:**
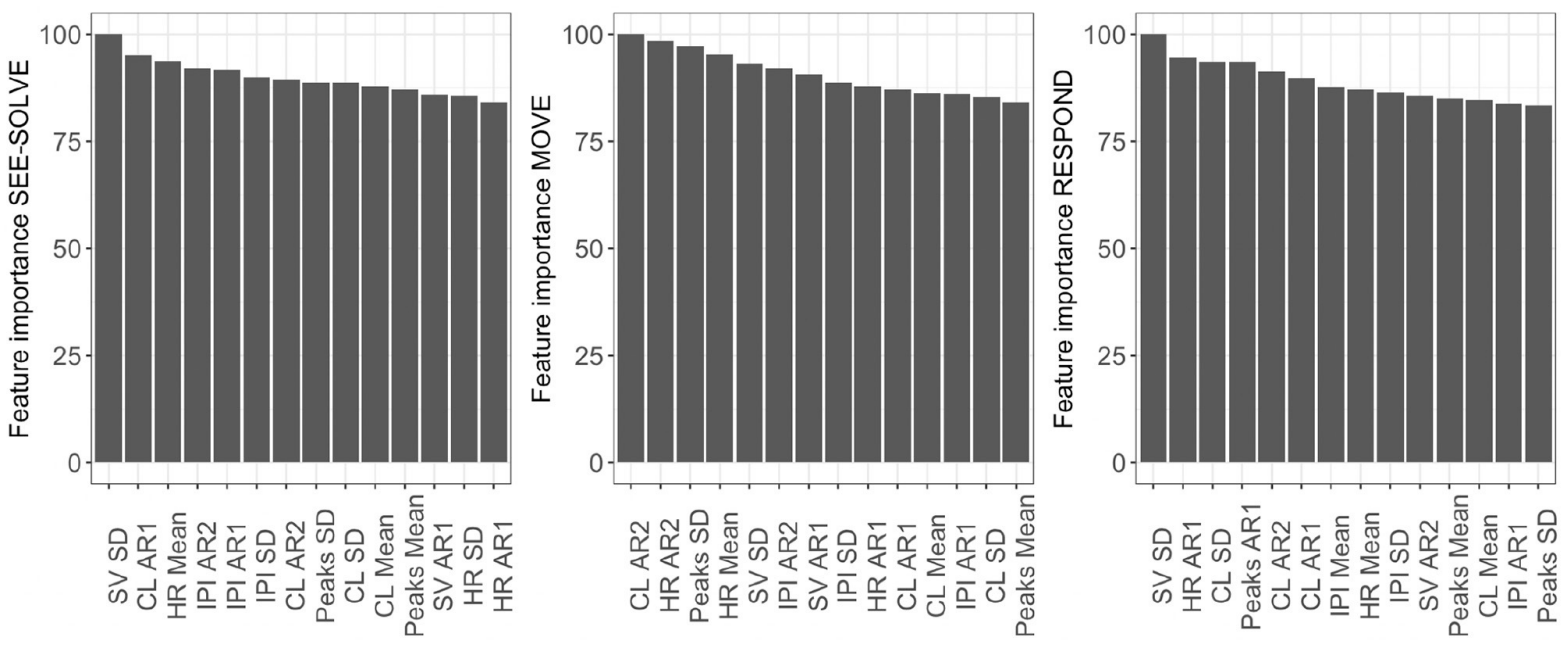
Variable Importance for predictive modeling using data from the different phases in Suffizz.

Considering the CFE from the predictive modeling for Suffizz, we observe that cognitive load mean, HR, mean and IPI SD, and first auto-correlation coefficient (AR1) demonstrate **Perfect CFE** because their individual feature importance ranks are the highest in See-Solve, followed by Move, and then the Respond phases (Figure 14 and Table 4). The set of features that demonstrates **Pseudo CFE** contains: first AR coefficient for cognitive load, mean number of EDA peaks, and saccade velocity SD. This is because these features have their highest individual feature importance rank in the See-Solve phase, but their individual feature rank in the Move phase is smaller than in the Respond phase (Figure 14 and Table 4). The remaining features do not exhibit CFE for either of the following two reasons: (1) they do not appear in the top 10% of the most important feature list for any phase of the S2MR cycle, or (2) their highest individual rank does not occur in the See-Solve phase.

## 6. Discussion

In this contribution, we present a study that investigates the relationship between students' MMD measurements and their learning performance (i.e., RQ1) and how these relationship can inform the design of a physio-cognitive aware agent (i.e., RQ2). We addressed RQ1 using inferential statistics and predictive modeling. We presented the concept of CFE and used the design implications of CFE to address RQ2. In this section, we discuss the results connected with the CFE, with a focus on its generalizability. Then, we present an interpretation of the results through the lens of RQ1, followed by design guidelines based on CFE as a response to RQ2.

### 6.1. Generalizability of the CFE

We observe different classes of CFE (perfect/pseudo), to be present in both the games, Sea Formuli and Suffizz. The CFE results clearly demonstrate a considerable overlap in the measurements that exhibit the CFE. However, certain specificities across the results between the two games are present. First, according to inferential results, IPI and heart rate demonstrate a Perfect CFE for both games; saccade velocity shows Pseudo CFE for the Sea Formuli and Perfect CFE for the Suffizz; finally, the number of EDA peaks contains Perfect CFE for Suffizz, but no CFE exists for Sea Formuli. Second, based on predictive modeling, Perfect CFE was exhibited by mean heart rate, mean cognitive load, and IPI (SD and first auto-regression coefficient). Whereas, saccade velocity SD shows Pseudo CFE for both the games.

For Sea Formuli, the collection of measures with CFE contains HR AR1 (Pseudo), mean phasic EDA (Pseudo), and CL AR2 (Pseudo); while for Suffizz it contains mean number of EDA peaks (Pseudo) and CL AR2 (Pseudo). These results show three levels of generalizability regarding the CFE: (1) CFE exists in the games with the different interactive elements and different domains; (2) some MMD measurements show the same CFE across both games (i.e., heart rate, IPI, and cognitive load, but with different effect sizes); and (3) there are some MMD measurements that show CFE but have different classes for different games (i.e., perfect in one game and pseudo in other). The results also reveal game-specific CFE. However, additional experiments are required to conclude if these specificities are due to interaction or domain differences between the games.

### 6.2. Interpretation of Results: RQ1

In this study, we captured and analyzed students' MMD as they problem-solved mathematics and language questions offered by two different MBEG. The MMD included physiological and eye-tracking data. We devised a novel index called CFE that illustrates the explainability (based on inferential statistics) and the prediction ability (based on predictive modeling) of the measurements/features, extracted from MMD, in terms of student's learning performance.

In both games, during the See-Solve phase, each of cognitive load, IPI, saccade velocity, mean HR, number of EDA peaks, and mean phasic EDA have a significant relationship with students' performance. The first three measurements (cognitive load, IPI, saccade velocity) were extracted from eye-tracking data, while the remaining three were derived from the Empatica E4 wristband data. Further, these eye-tracking variables were significantly lower for the right responses than for the wrong responses. According to previous research, high cognitive load is detrimental to learning outcomes/performance (De Neys and Schaeken, 2007; Feldon et al., 2018; Mutlu-Bayraktar et al., 2019). Cognitive load, as measured by eye-tracking data, contains an interaction of intrinsic, germane, and extraneous components (Paas et al., 1994; Sweller et al., 1998). The intrinsic components include the proficiency and mental models of the students. The extraneous components include the content and its presentation. Finally, the germane component results from the interaction between the student and information (Paas et al., 1994; Sweller et al., 1998). In each case, guiding students' attention to specific parts of the screen (Jarodzka et al., 2010) and providing worked examples (Van Gog et al., 2015) might have a positive relationship with the student's cognitive load and learning performance. It is important to point out here that, we are not attempting to keep the cognitive load to a very low level. We are proposing remedial actions for the prevention of cognitive-overload (for which we do not have a measurement) by avoiding very high cognitive load values.

Additionally, in both MBEG, saccade velocity was higher for the interactions associated with wrong responses than for the interactions associated with right responses. Previous eye-tracking research has shown saccade velocity to be related to task complexity and perceived task difficulty (Smit and Van Gisbergen, 1989; Schubert and Zee, 2010). We offer two plausible explanations for these two events (e.g., students having high saccade velocity and providing wrong responses), coinciding in a significant manner. First, the question content might be too complicated for students; and second, the students perceive the problems as difficult to solve. In both cases, performance is hindered, and in both cases, introducing guiding feedback is necessary to improve students' task-based proficiency (Lipp and Hardwick, 2003).

The final eye-tracking measurement, IPI, also demonstrated significant differences between right and wrong responses for both MBEG. Recall that IPI is the ratio between global and local processing (Unema et al., 2005; Poole and Ball, 2006). A significantly higher IPI during interactions associated with wrong responses indicates that global processing is higher when students encounter a problem that they are unlikely to solve (i.e., provide a wrong response). This suggests that students are unable to properly manage their attention span when they are faced with challenging questions for which they are unlikely to solve correctly (Tsai et al., 2012). An additional explanation for higher global processing could be that the students are looking back and forth between the options and the question and between the options themselves. Such patterns are indicative of guessing behaviors and are often encountered in learning systems with multiple choice questions and quizzes (Tsai et al., 2012). In such cases, attention and/or strategy based feedback might assist the students (Collignon et al., 2020).

Concerning the physiological measurements, during the See-Solve phase in both MBEG, the mean HR was significantly higher for wrong responses than the right response. Higher mean HR indicates higher stress levels (Harada, 2002; Herborn et al., 2015), which have been shown to be detrimental to learning performance (Sharma et al., 2019a,b). The significant relation between wrong responses and mean HR illustrates that students experience higher stress levels when they provide a wrong response. Consequently, in such cases an affective intervention (e.g., removing time constraints or pausing the game) could help the students re-establish their performance levels (McCraty, 2005).

Furthermore, for both MBEG, the number of EDA peaks and the mean phasic EDA levels were higher in cases of wrong responses than right responses. High levels of phasic EDA and a high number of EDA peaks are correlated with higher emotional arousal (Di Lascio et al., 2018) and found to be negatively correlated to students' learning outcomes/performances (MacLean et al., 2013). Thus, in these cases, feedback to regulate students' emotional tendencies should be employed, as previous research demonstrates this to be highly effective in scaffolding the problem-solving processes (Lipnevich and Smith, 2009; Harley et al., 2019).

During the Move and Respond phases of the S2MR cycle, we observed a collection of counter-intuitive MMD-based differences. For example, IPI, saccade velocity, mean HR, and the number of EDA peaks were each significantly different for right and wrong responses. Moreover, these unexpected differences all share the same affinities (though, with lower effect sizes) with the students' performance levels. Specifically, IPI, saccade velocity, mean HR, and the number of EDA peaks had an affinity with the wrong responses, indicating that these measurements have detrimental effects on learning performance. Interestingly, because no problem-solving takes place during these phases, there is no justifiable basis for these effects to occur. However, these results reveal that for phases unrelated to the problem-solving aspects of the MBEG interaction (i.e., Move, Respond), students continued to display behavioral patterns which had affinity with the wrong responses.

These aforementioned behavioral patterns (IPI, saccade velocity, mean HR, number of EDA peaks) exhibit the **CFE**. The basic concept behind the CFE is that the effect sizes “carry forward” to subsequent phases of S2MR, and diminish in size along the way. The measurements that demonstrate the CFE (i.e., perfect or pseudo) are decisive for the current task and also the forth-coming tasks in following phases. If a measurement has an affinity with the desired outcome (e.g., the right response in our case), then the CFE should be promoted, otherwise (i.e., if the measurements have an affinity with the undesired outcome), remedial steps should be taken to reduce/terminate the negative consequences of this chain of behavioral patterns.

Our inferential statistics showed that four measurements exhibit CFE (perfect or pseudo): IPI, saccade velocity, mean HR, and number of EDA peaks. Predictive modeling associated CFE (perfect or pseudo) with the following: fixation duration (SD and auto-regression), cognitive load (mean, SD), HR (mean and auto-regression), and mean phasic EDA. Moreover, there is considerable overlap in the basic measurements (refer to Table 2, except cognitive load) in these two sets^3^, and these results hold true for both MBEG (as discussed in section 6.1). Therefore, we continue with the basic measurements (i.e., cognitive load, IPI, saccade velocity, HR, phasic EDA, and the number of EDA peaks) for the remainder of this discussion. **Lastly, a critical commonality shared by these measurements is the affinity with the wrong response. Hence, in the context of this study, we discuss design of an AI agent that mitigates these detrimental effects**.

It is important to point out that what is detrimental is neither the measurement nor the CFE. The detrimental fact is that the measurement displays CFE. For example, if students are showing moderate stress and at the same time their responses are correct, there is no requirement of remedial action. The reason is that we are measuring stress and not chronic stress. Remedial action is required in the cases where a high stress level has corresponding incorrect responses. We propose that the requirement of such remedial action is elevated in the cases where the stress is not only related to wrong responses but also showing CFE. Moreover, CFE presents an approach to prioritize the MMD measurements in the following order. (1) If the measurement shows perfect CFE and is associated with the incorrect response. (2) If the measurement shows partial CFE and is associated with the incorrect response. (3) If the measurement is associated with the incorrect response.

While CFE provides an approach to prioritize the scaffolds, the key factor in deciding the scaffold is the factor that is negatively related to the problem-solving process (e.g., lack of knowledge, low self-efficacy). These factors, in turn, are related to certain behavioral aspects (e.g., lack of knowledge could lead to cognitive load, especially the intrinsic cognitive load and low self-efficacy could lead to stress). MMD-based measurements provide a proxy to these factors (e.g., pupil diameter for cognitive load and increase in heart rate for physiological stress).

#### 6.2.1. CFE From Statistical and Predictive Standpoints

The main reason for using both the statistical and predictive modeling of the data was to showcase that CFE can be established between measurements and learning performance (i.e., the correctness of the responses in our case) using any method that provides an estimate of the models' quality. In our examples, there are considerable similarities based on the measurements that we used for statistical models and the measurements that were used for extracting features in the predictive modeling. Information processing index, heart rate, saccade velocity, and the number of EDA peaks are the measurements that show one kind (either perfect or pseudo) CFE in both statistical and predictive modeling. These measurements and the related implications are in the next subsections. However, there are certain differences in the results from the two modeling approaches as well. First, the cognitive load does not show any CFE in the statistical modeling while the mean and autoregressive coefficients of cognitive load show CFE in the predictive modeling. Second, phasic EDA also shows CFE using predictive modeling, while it does not show any CFE using statistical modeling. Another set of differences in the results from these two modeling approaches is in the strength of CFE. Both these differences could be attributed to the fact that the statistical approaches used in this paper assume linear relationships between the variables, whereas there is no such assumption in the predictive modeling. Moreover, with some prediction algorithms, we are looking for non-linear relations (e.g., support vector machines and Gaussian process modeling with polynomial kernels). It has also been argued that both extremes of cognitive load are detrimental to learning performance (Czikszentmihalyi, 1990; Collins et al., 1991), indicating a non-linear relationship. Another reason for using these two modeling approaches in the paper is to provide both ways of inferring CFE. The benefit of using statistical modeling is the opportunity to find direct relationships between the dependent and independent variables. On the other hand, the benefit of predictive modeling is the ability to utilize non-linear relationship and stronger quality measurements than statistical modeling.

We have carried out the two analyses to showcase that the CFE can be established using either the inferential statistical methods or the predictive modeling methods. The main idea is to show the compatibility of the effect with the two sets of methods. The decision about which one of the two methodologies to be used, completely depends on the availability of the data and level of explanation power required appropriate by the researcher.

### 6.3. Toward a Physio-Cognitive Aware Intelligent Agent to Support Learning (RQ2)

We distilled a set of MMD measurements that demonstrate CFE (Perfect or Pseudo), relative to the students' level of correctness (right or wrong). In this section, we offer a collection of design guidelines motivated by the behaviors of these variables. Additionally, we provide a feedback agent design comprised of the aforementioned guidelines and a decision-making protocol that prioritizes the type of feedback (attentional, cognitive, affective) to provide students during the different phases of their MBEG play interaction. While designing an MMD-based intelligent agent, in case of conflicting recommendations, we prioritize recommendations based on the variables in the following class order: Perfect CFE → PseudoCFE → No CFE. In the case of a tie, variable importance (in the terms of predictive power with respect to the dependent variable) and/or the effect size of the variable will be the deciding factor.

The following design of an agent is one of the primary implications of the results of these studies. The main idea here is that we are proposing a method to prioritize the feedback (in the cases it is necessary). The main discussion is about the prioritization in this subsection. The type of feedback is inspired from the related research and to prove that such a system works, further development and testing are necessary. Moreover, our results show that there are significant relations between the multi-modal measurements and the correctness of the students' responses. In the light of these results, we are proposing certain implications for interventions. These individual interventions have been shown to be able to help students in improving their learning performance and learning experiences.

#### 6.3.1. Measurements to Feedback

If a measurement has an affinity with the right response (or more generally, if it exhibits affinity with positive outcomes and performance variables), remedial action is not needed. However, it is important to provide students with feedback, such as encouragement, as positive re-enforcement has been linked to positive effects on students' task-based outcomes/performance and self-esteem Helm (2007). Contrarily, when a measurement has an affinity with the wrong response, there is a need for remedial action in order to counter the CFE.

In the remainder of this subsection, we present mechanisms that have been found to be effective in terms of reducing the impact of adverse cognitive and physiological behavior. Among these MMD measurements, three were derived from eye-tracking glasses (i.e., cognitive load, saccade velocity, IPI), and two from physiological sensors (i.e., HR and EDA).

First, if a student's cognitive load is high (e.g., negative affinity with performance), there are several methods/strategies that can be integrated into an AI agent, which reduce cognitive load so that it is no longer detrimental to the student's productivity. For example, the AI agent can present a related solved example problem, or provide content related hint to help the student solve the problem correctly. Alternatively, the agent could scale down the question difficulty, by providing an easier (but related) problem to solve. This provides the student with an opportunity to practice (and internalize) the target concepts, and build self-assurance, before increasing the complexity of the problems at a later stage. It is important to point out here that we do not aim to reduce cognitive load to zero, our aim is to keep the cognitive load at a manageable level so that we minimize the probability of cognitive overload (which we do not have a measurement for). Therefore, we want to avoid higher values of cognitive load.

Second, we consider periods where a high saccade velocity (indicating high perceived task difficulty/complexity) is detected. In this scenario, the problem content might not be difficult given a student's expertise, however, they still may perceive it as such. Correspondingly, a small hint might help a student solve the problem correctly while providing them with additional “confidence” for the future problems. Alternatively, if the given problem is difficult for a student to solve (e.g., beyond their knowledge set or cognitive capabilities), a content-based hint may assist them, and prepare them for upcoming problems. If the AI agent has an estimate of the student's expertise in the given domain (e.g., from their responses to previous questions), then the AI agent can choose between these two feedback options; that is, small hint or solved examples.

Concerning the last eye-tracking measurement, IPI may require counter-active measures to prevent or deter CFE. Specifically, we propose the use of gaze-contingent support (Sharma et al., 2015) when considering feedback for increasing the local processing (i.e., decreasing IPI) or the global processing (i.e., increasing IPI). Gaze-contingent interfaces overlay on-screen content with a domain expert's gaze (Jarodzka et al., 2010). Gaze-contingent is useful in explaining different concepts to students and novices as the overlays illustrate how an expert's gaze traverses the interface while the expert solves a similar problem (Jarodzka et al., 2010; Van Gog et al., 2015). This approach has the capacity to help the students manage their attention (and in turn information processing behavior) in a manner that might increase the probability of correct responses.

The final two measurements, EDA and mean HR, derive from physiological sensing (e.g., the Empatica E4 wristband) and are, respectively, related to the student's physiological arousal and physiological stress. To deter increasing mean HR (often indicative of high stress levels and in our case, associated with a negative affinity with performance), the agent should suggest that the student take a short pause from the learning activity, so they can relax before resuming the activity under a lower stress level. Stress can have a negative impact on the performance, as was found in our results. Therefore, suggesting a small pause might reduce stress, and increase the probability of bringing students closer to a state that students' experience when optimally engaged in an activity (i.e. “flow” state) (Czikszentmihalyi, 1990) and reinforce their abilities to be able to respond correctly in the future problems.

#### 6.3.2. Type and Timing of Feedback

**Figure 15:**
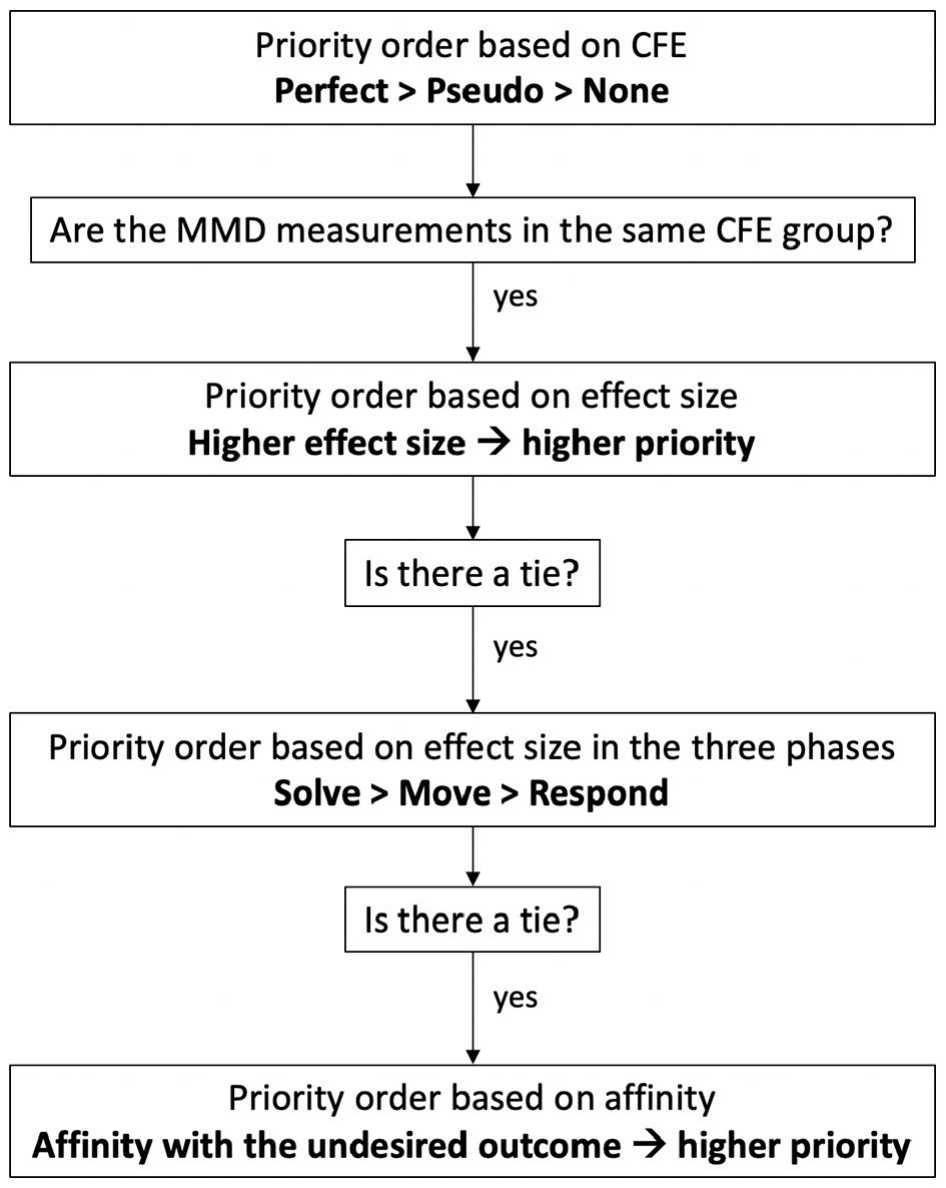
Prioritizing the MMD measurements based on CFE.

In the previous section, we presented which type of feedback to deliver to students, according to the different measurements computed from the MMD. In this section, we define how the CFE can be used to prioritize this feedback (Figure 15 shows the summary). Prioritized decision making is useful for supporting two scenarios. First, the gameplay session structure (i.e., time or space resources) may only be able to accommodate single feedback delivery. Second, the delivery of various and, at times, conflicting feedback (e.g., saccade velocity suggests an easier problem based on perceived difficulty and EDA suggests a more difficult problem based on arousal; or one feedback suggests positive enforcement and the other feedback suggests corrective) could result in students' disengagement from the MBEG or hinder the student's state of “flow.” Following, we discuss the prioritized order for the types of feedback based on the class of CFE present.

We claim that CFE possesses an innate priority ordering that dictates the sequence in which to address MMD measurements with feedback delivery. The highest priority is given to measurements which exhibit Perfect CFE, followed by Pseudo CFE, and lastly, no CFE. The rationale behind this prioritization assignment is that the CFE propagates to phases where it is “undesirable” and therefore, should be prevented or subdued. Thus, we propose a CFE priority order of Perfect, Pseudo, None. In our case (using our set of MMD measurements), the resulting order is mean HR or IPI, followed by saccade velocity, followed by all remaining measurements listed in Table 2.

However, each CFE type contains MMD measurements, which may also lead to different (or worst case, conflicting) feedback suggestions. For example, the feedback suggestions for mean HR and IPI might suggest the student take a pause to reduce physiological stress, and show an expert's gaze patterns to manage the student's visual attention. Correspondingly, further prioritization is needed within the aforementioned CFE type prioritization. Additionally, as previously mentioned, there might only be time for delivery of one feedback mechanism, or it may not be possible (or purposeful) to deliver multiple feedback options in tandem (e.g., it is counter-productive to show the students the expert's gaze patterns during a pause). Therefore, within CFE type prioritization, we guide the decision-making process by the effect sizes of the given MMD measurements, in terms of their affinity with the dependent variable.

In our dataset, mean HR has a higher effect size (0.31, 0.21, and 0.16 for the See-Solve, Move, and Respond phases, respectively) than IPI (0.22, 0.16, and 0.12 for See-Solve, Move, and Respond phases, respectively). Therefore, we assign the mean HR with a higher priority than IPI. Applying this rule to the remaining MMD variables results in the following priority order: mean HR, IPI, saccade velocity, and all remaining measurements from Table 2.

Once MMD measurements have been prioritized according to their CFE type and effect size, the variables with no CFE remain. We propose a two-step process to prioritize these variables: (1) the order of the significance as See-Solve > Move > Respond; (2) higher effect sizes take precedence. For example in Sea Formuli, phasic EDA (which is significantly different between levels of correctness during see-solve and move phases) had a higher priority over cognitive load (significantly different between the correctness levels during the see-solve phase). However, for Suffizz, Cognitive load (0.45 effect size in See-Solve phase) also takes precedence over phasic EDA (0.34 effect size in See-Solve phase). Applying this strategy to the MMD measurements from Table 2 result in the following priority order: mean HR, IPI, saccade velocity, cognitive load, phasic EDA, and the number of EDA peaks.

Finally, if the above criteria are identical for two MMD measurements, the measurement with affinity to an undesired outcome variable (e.g., performance, engagement) is assigned priority over the measurement with affinity to the desired outcome variable. Our working hypothesis suggests that this case is rare and thus, ranking measurements in their order of preference for feedback suggestions will not be needed. For example, we do not need to use this as a tie-breaking rule for the MMD measurements used in this contribution.

### 6.4. Theoretical and Practical Implications

In this contribution, we present a special phenomenon CFE that indicates toward certain physio-cognitive measurements having extended effects on the learning experiences and performances than the others. CFE could be used by educators and learning-technology designers to understand and develop scaffolding tools to support learners in GBL settings. As aforementioned, CFE provides the sequence in which to address multimodal measurements while supporting the learners. Such a priority sequence is important when the time for support is short or the frequency of supporting cues should be low. Tropper et al. (2015) suggested that the scaffold should be adaptive, dynamic, and fading (should be provided fewer times as the learners' interaction with the environment becomes longer). With CFE, it is possible for scaffolds to gradually “fade-away” by providing the feedback with the highest priority. CFE can also be used in more generalized scenarios than GBL. For example, in Intelligent Tutoring Systems (ITS), where there are clear steps/phases to complete the given task. One of the recurring problems in ITS and cognitive tutors is the “assistance dilemma” (Koedinger and Aleven, 2007; McLaren et al., 2014), which is the requirement of the trade-off between the timing of providing the feedback and the amount of feedback provided. Excess feedback might be detrimental to learning performances and experiences (Koike et al., 2021). At the same time, supporting students at the wrong time could drastically increase their cognitive demands (Schwartz and Bransford, 1998) and also have negative impacts on their task performance and affective states (Hattie and Timperley, 2007; Wisniewski et al., 2020). CFE might be helpful in the situations where the amount of feedback requires regulation by suggesting the most appropriate feedback for the moment.

As we mentioned in section 1.1 that in both problem-based and GBL settings, it is important to provide the learners with dynamic and adaptive scaffolding (Quintana et al., 2004; Leemkuil and Jong, 2011). The combination of the CFE and the see-solve-move-respond phases could be an automatic and data-driven solution for the “assistance dilemma” (Miwa et al., 2012; Maniktala et al., 2020). It is important to provide a timely and appropriate amount of feedback to the learners (Li et al., 2018) and if we know the problem-solving phase the learner is in and the priority list of the feedback options, we can optimize the learning experience by controlling multiple feedbacks (Li et al., 2018). It is also important to understand the constraints of the interactive situation before providing the feedback (Khodeir et al., 2018), which can be achieved by having the CFE-based priorities a way to optimize the constraints-solution. Furthermore, CFE can be considered an addition to the existing learner models. There have been individualized sensor data added to pre-existing learner models. For example, affective states (Grawemeyer et al., 2017; Rajendran et al., 2018), and eye-tracking based measurements (Njeru and Paracha, 2017). CFE can provide a way to combine data and measurement from multiple sensors in a single learner model by using the priorities as weights in the model and to predict/understand the learners' needs in a better manner.

One of the key considerations emerging from this contribution is that the performance measure used was a dichotomous correct/incorrect division. The choice of the learning performance measure and/or the learning experience measurement might have an impact on the findings. For example, if the measure of interest is not the performance but it is the skill-acquisition or comprehending the problem solving processes. In such a case, the CFE would have to be considered using the multimodal measurements that would correspond to and be associated with “lack of” acquired skill and “poor understanding” of the problem solving process. Similarly, the see-solve-move-respond cycle will have to be altered to reflect the correct phases of the underlying tasks. Moreover, in cases where the performance measure is more complicated than a dichotomous correct/incorrect marking, the complexity and nuances of the performance measurement would have to be taken into account. For example, if the learning task is a synthesis task (e.g., concept-mapping), then the various factors of a successful synthesis should be considered (e.g., understanding, evaluation, and transfer in the case of concept-mapping). In such cases, the multimodal measurements would have to be examined against an individual component of the performance measurement, which might result into a complex model (i.e., difficult to implement).

In this contribution, we have combined eye-tracking data with heart rate and EDA to define various learning constructs. For example, cognitive load (pupil data), engagement (EDA), and stress (heart rate). The main idea is to combine/fuse them in a manner so that we can not only detect different thematic phases from the interaction but also provide meaningful and actionable feedback to the learners. This is inline with the contemporary research using the MMD for improving understanding and design of educational technology (Giannakos et al., 2019; Liu et al., 2019; Sharma et al., 2019a; Lee-Cultura et al., 2020b). The measurements from different data sources could also be used to indicate a broader learning construct. For example, pupil diameter, heart rate, and number of EDA peaks could be used to define a new multimodal measurement of physio-cognitive stress/load while solving educational problems. Recent research has shown that fusing the data together results in better models in the learner-technology environment (Giannakos et al., 2019; Liu et al., 2019) but whether combining measurements from the different sources would offer a better understanding of the underlying phenomenon is yet to be seen and therefore more research is required in this direction.

When it comes to the scaling-up aspects of the CFE, our contribution could be extended by only using data from the sources that are available in a ubiquitous manner. Following the same process as to find the CFE with other data sources would provide the priority order of the measurements at hand. This would help the educators and designers create seamless and at-scale scaffolding systems (e.g., using data from a smart watch and webcam to capture hear rate and facial expressions, respectively). The see-solve-move-response cycle could also be extended to any other situation where the problem-solving steps can be detected as phases and the data could be collected from the individual phases. Recently, in related fields such as UbiComp, there have been approaches to scale-up the sensing using mobile and pervasive data-sources (Visuri et al., 2018; Wang et al., 2019). We believe that our work could be scaled up with such techniques and with contextual awareness (tracking the performance for a given task, Hossain and Roy, 2019), ambient intelligence (using multiple sensors in a setting, Giannakos et al., 2020), and monitoring/tracking students to provide them support in a seamless manner (Weiser and Brown, 1997). However, there might be separate practical and ethical concerns with exploring the appropriate scale to which CFE can be extended.

### 6.5. Limitations and Future Work

This contribution has a practical and theoretical impact on the embedding AI within educational technologies, however, there are limitations that must be addressed for further improvement. For example, we are determine if cognitive load or stress induced during MBEG play sessions, originated from the problem-solving task or the novelty of interaction (although, the children were given a few rounds to play the games so that they can get acclimatized with the learning environment). To accommodate for this, future work will include a longitudinal study to reduce the novelty of the interaction, and so we can assess the root cause of students' cognitive load and stress. Next, although we altered the problem content to align with the students' projected abilities (as defined by their year of study), a different age range (other than 9–12) may have yielded different results. Moreover, in our approach, we used a particular measurement to portray students' performance (i.e., the correctness). This measurement is widely used; however, using a different measurement as the dependent variable may yield different results. Thus, although we followed an ecologically valid and accurate research design, we acknowledge that other methodological decisions might play an important role in the results. Future avenues emerging from this work is implementing a realized system that adheres to the design guidelines and conducting a study to determine its efficacy (relative to students' performance). Additional work stems from the fact that this study has utilized state-of-the-art sensing equipment with high quality data collections. An interesting challenge of future studies is the need to consider the feasibility and performance of MMD collected from widely used sensing technology (e.g., estimating eye-tracking *via* cameras, more affordable wristbands). Solving such a challenging engineering problem will pave the way for democratizing this technology and allow individuals and society to leverage physio-cognitive aware learning systems. Another avenue for the future work would be to combine the low-level multi-modal measurements into high-level constructs to obtain a more holistic understanding of the learning processes in GBL. This could be made possible by understanding the interrelations among the low-level measurements and then exploring their relations with various measures of learning performance and learning experiences. Finally, the individual characteristics of the learners (e.g., motivation, strategy, attitudes) could impact the relationship between the multimodal measurements and the learning performance. Another future aspect of this work is to examine how such variables moderate/mediate the relations presented in this contribution.

## 7. Conclusion

In conclusion, we presented a study with 40 students playing two MBEG where their MMD (eye-tracking, HR, and EDA) were recorded. Using both inferential statistics and predictive modeling, we defined CFE with respect to the correctness of the students' responses (right or wrong). We deduce that the notion of CFE plays a vital role in the design of feedback/support to be used in an intelligent agent to support students based on their personal MMD measures. Our results show that HR and information processing behavior measurements require the most attention. However, these CFE-based findings require further experimentation for generalization, and there is a need to further explore the CFE with MMD to establish a generalized theoretical framework.

## Data Availability Statement

The datasets presented in this article are not readily available because due to the ethics regulations we can not share the data. Requests to access the datasets should be directed to kshitij.sharma@ntnu.no.

## Ethics Statement

The studies involving human participants were reviewed and approved by Norwegian Center for Research Data (NSD). Written informed consent to participate in this study was provided by the participants' legal guardian/next of kin.

## Author Contributions

KS: conceptualization, data collection, methodology, and writing–original draft. SL-C: conceptualization, data collection, and writing–original draft. MG: conceptualization and writing-original draft. All authors contributed to the article and approved the submitted version.

## Conflict of Interest

The authors declare that the research was conducted in the absence of any commercial or financial relationships that could be construed as a potential conflict of interest.

## Publisher's Note

All claims expressed in this article are solely those of the authors and do not necessarily represent those of their affiliated organizations, or those of the publisher, the editors and the reviewers. Any product that may be evaluated in this article, or claim that may be made by its manufacturer, is not guaranteed or endorsed by the publisher.

## Footnotes

^1^In this paper, the learning performance is measured by the correctness of students' responses to the in-game questions.

^2^The rank of the feature is determined by the fact that how important it is in the prediction of the dependent variable as compared to other features.

^3^IPI is a Pseudo function of fixation duration.
